# Trends in ICU mortality and underlying risk over three decades among mechanically ventilated patients. A group level analysis of cohorts from infection prevention studies

**DOI:** 10.1186/s13613-023-01159-0

**Published:** 2023-07-11

**Authors:** James C. Hurley

**Affiliations:** 1 Melbourne Medical School, University of Melbourne, Melbourne, Australia; 2 Division of Internal Medicine, Grampians Health, Ballarat, VIC Australia; 3 Internal Medicine Service, Ballarat Health Services, PO Box 577, Ballarat, 3353 Australia

**Keywords:** Mortality, Infection prevention, Decontamination, APACHE II

## Abstract

**Background:**

Has either the underlying risk or the mortality incidence among ICU patients receiving mechanical ventilation (MV) in the literature changed in recent decades? Interpreting ICU mortality trends requires an adjusted analysis accounting for changes in underlying patient risk.

**Methods:**

Control and intervention groups from 147 randomized concurrent control trials (RCCT) of various VAP prevention interventions, as listed primarily within 13 Cochrane reviews and 63 observational studies listed﻿ primarily within four systematic reviews. Eligible studies were those including ICU patients with > 50% of patients receiving > 24 h of MV with mortality data available. ICU mortality (censored day 21 or before) or late (after day 21) mortality together with group-mean age, and group-mean APACHE II scores were extracted from all groups. These incidences were summarized in five meta-regression models versus publication year being variously adjusted for age, APACHE II scores, type of study intervention and other group level parameters.

**Results:**

Among 210 studies published between 1985 and 2021, 169 being found in systematic reviews, the increase per decade in mean mortality incidence, group-mean APACHE II scores, and group-mean age, were < 1 percentage point (*p* = 0.43), 1.83 (95% CI; 0.51–3.15) points, and 3.9 (95% CI; 1.1–6.7) years, respectively. Only in the model with risk adjustment for both group-mean age and group-mean APACHE II score was a significant decline in mortality apparent. In all models, the mortality incidence among concurrent control groups of decontamination studies was paradoxically five percentage points higher than benchmark and showed greater dispersion.

**Conclusion:**

Mortality incidence has changed little over 35 years among ICU infection prevention studies whilst the patient age and underlying disease severity, measured as APACHE II, have both increased. The paradoxically high mortality among concurrent control groups within studies of decontamination methods of infection prevention remains unaccounted for.

**Supplementary Information:**

The online version contains supplementary material available at 10.1186/s13613-023-01159-0.

## Key points

- **Question**: Has ICU mortality incidence among studies in Cochrane reviews of infection prevention interventions for mechanical ventilated patients changed over three decades or has the underlying disease severity, measured as APACHE II and age, changed?
- **Findings**: Among broad categories of control, intervention, and observational groups, the trends in mortality incidences are similar, except for control groups within studies of decontamination interventions which paradoxically, show higher than expected mortality.
- **Meaning**: A decline in ICU mortality over three decades becomes apparent only after adjusting for both age and underlying risk measured as APACHE II.

**Supplementary Information:**

The online version contains supplementary material available at 10.1186/s13613-023-01159-0.

## Background

Both disease severity at the time of ICU admission and whether infection is acquired following admission are key determinants of the prognosis of ICU patients [1, 2]. Whether prognosis has improved in recent years with, on one hand, aging of ICU patient populations and, on the other, with infection prevention interventions applicable to patients receiving or likely to receive mechanical ventilation (MV), are two research questions of great interest.

Studies of infection prevention interventions among MV patients published over three decades provide group level measures of disease severity, measured as APACHE II and age, and outcome data, as ICU mortality, for patients requiring prolonged ICU admissions. In considering whether the data from these studies could form the basis for addressing these questions, five aspects require consideration. First, the validity of illness severity scores developed in the early 1980’s to mortality observations collected up to four decades later remains to be established.

Second, as the upper age breakpoint within the APACHE II score is age 75 years, this score may underestimate the impact of age > 75 years on mortality risk in the current era [1].

Third, infection prevention interventions, if successful, may have impacts on both the population risk, as herd effects, as well as the individual risk [3–5]. The potential for herd effects was recognized in the first study of Selective digestive decontamination, being antibiotic-based decontamination using topical antibiotic prophylaxis (TAP) [6]. By design, this study, and several since, deliberately used non-concurrent control groups to provide external benchmarks immune from any potential spill-over effects of the intervention on the study outcome on bystander patient groups within the ICU population [7–9]. Hence, control groups within randomized concurrent controlled trials (RCCT) versus cluster randomized trials (CRT) of decontamination studies might provide different incidence estimates.

Fourth, completeness of patient follow-up, a key attribute of RCCT’s, needs to be established to ensure that valid mortality estimates have been reported.

Finally, the analysis needs to consider whether the patients and the studies within different categories of systematic reviews are representative of the broader published literature beyond RCCT’s.

The primary aim here was to assess trends over time in ICU mortality and measures of underlying risk, measured as APACHE II and age, using study data abstracted in systematic reviews of infection prevention interventions and the broader literature. The secondary aim was to compare mortality trends among studies of decontamination methods, which overall appear generally more successful at infection prevention versus non-decontamination methods. To this end, a comparison category of observational studies without a study intervention is required to provide an external benchmark for each measure.

## Methods

This analysis is based on studies of interventions to reduce the incidence of ventilator associated pneumonia (VAP) and other infections in patients who are receiving or have the potential to receive prolonged MV in ICU.

The literature search here is opportunistic in that the studies were primarily sourced from Cochrane and other systematic reviews of interventions that could be used to prevent infections and mortality in MV patients. I searched the Cochrane library from Jan 1, 2012, to Dec 7, 2021 for systematic reviews of infection prevention interventions applicable to patients at risk of acquiring infections whilst receiving prolonged mechanical ventilation whilst in the intensive care unit [10–26]. I used search terms related to the prevention of infection, whether decontamination-based or non-decontamination-based methods and applicable to patients receiving MV. Systematic reviews limited to interventions applicable to specialized populations, such as paediatric or patients with Adult respiratory distress syndrome (ARDS), were excluded. Since systematic reviews generally include only RCCT’s the search was supplemented by a search for CRT’s of methods of ICU infection prevention. CRT’s and additional systematic reviews and meta-analyses were identified using the literature as cited by the most recent CRT [9] and systematic review [27] together with a search using the related article function in Google scholar.

The literature search here modifies earlier search criteria by limiting inclusion to those studies which reported the underlying disease severity using the APACHE II score [28].

These systematic reviews were each searched for studies meeting the following inclusion criteria; published after 1985, patient populations for which > 50% required prolonged (more than 24 h) ICU admission and MV; mortality incidence data and illness severity reported using the APACHE II score.

The following exclusion criteria were applied: studies limited to specific patient populations, such as those with ARDS, studies limited to patients post cardiac surgery and studies limited to the pediatric age group (< 18 years). Studies in languages other than English were included when the required data had been abstracted in an English-language systematic review.

Studies identified from using the “related studies” search that met the inclusion criteria were also included. All data and references together with a listing of the original studies are located in the Additional file 1 together with additional diagrams, tables, and figures).

### Study selection and decanting of groups

**Fig. 1:**
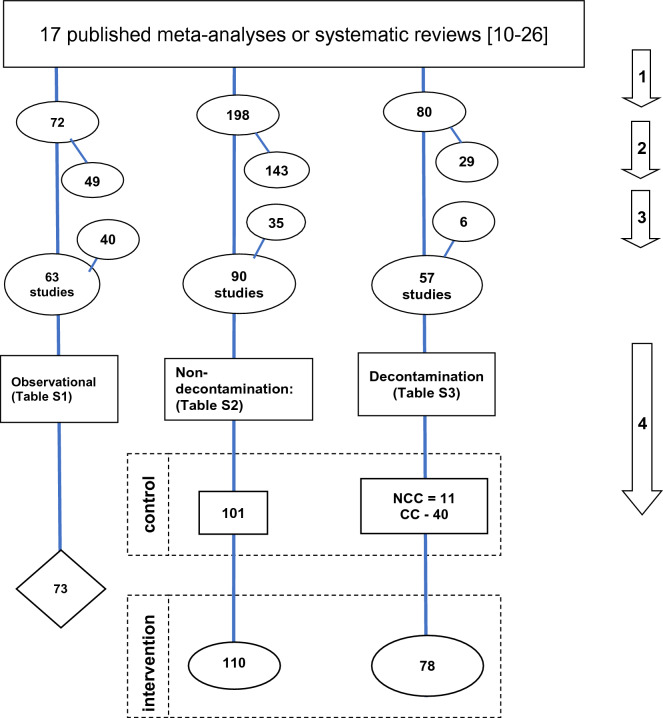
Search method, screening criteria and resulting classification of eligible studies and subsequent decant of component groups. The four numbered arrows are as follows; (1) An electronic search for systematic reviews containing potentially eligible studies using search terms; “ventilator associated pneumonia prevention”, “mechanical ventilation”, “intensive care unit”, each combined with either “meta-analysis” or “systematic review” up to December 2021 within The Cochrane database of systematic reviews. The systematic reviews were streamed into one of three categories; studies in which there was no intervention (observational studies), studies of various non-decontamination methods such as methods delivered either via the gastric route, the airway route or via the oral care route, studies of decontamination methods including studies with either an anti-septic or topical antibiotic (in any formulation)-based intervention. (2) The systematic reviews and meta-analyses were then searched for studies meeting the following inclusion criteria; (1) patient populations requiring prolonged (> 24 h) ICU admission; > (2) 50% of patients receiving mechanical ventilation for > 24 h; (3) mortality data available. (4) APACHE II score data available. And exclusion criteria; (1) Studies limited to paediatric ICU’s [mean age < 18 years]. (2) Studies limited to populations of ARDS patients (3) Studies limited to populations of Cardiac surgery patients. (3) Any duplicate or ineligible studies were removed and studies identified outside of systematic reviews, obtained by ‘snow ball sampling using the ‘related studies’ function in Google Scholar, were included. (4) The component groups were decanted from each study being control (rectangles), intervention (ovals) and observation (diamond) groups. NCC = non-concurrent control; CC = concurrent control. The total numbers do not tally as some systematic reviews provided studies in more than one category and some studies provided groups in more than one category. Also, some studies contribute both ICU and late mortality data

The literature search and analytic approach used here is detailed in Fig. 1. In brief, the studies were streamed into one of three broad categories as follows; methods based on the use of either TAP or topical antiseptics applied to the oropharynx or digestive tract to remove the colonizing flora (decontamination methods), methods based on either the gastric or airway-based interventions with the potential to reduce the risk of acquiring infection from the colonizing flora (non-decontamination methods), and studies without an infection prevention method under study (observational groups).

The component groups of the studies were decanted into strata of observational, control and intervention groups. The control groups were classified as either concurrent and co-located within the same ICU as the intervention group (concurrent control) or not (non-concurrent). The non-concurrent control groups were analysed as observational groups except where indicated.

For each group and each study, the key data were abstracted from the systematic review with the original study being reviewed for clarification in the case of any ambiguities.

### Outcomes of interest

Mortality, either as ICU mortality (censored day 21 or before) or late (after day 21) mortality, expressed as a proportion is the number of deaths with the total number of patients as the denominator. In addition, the following were also extracted where available; the proportion receiving MV, whether the ICU was a trauma ICU, being defined here as having > 50% of patients admitted for trauma, the group-mean (or median) age, the group-mean (or median) APACHE II score, year of study publication and whether the component group was exposed to protocolized parenteral antibiotic prophylaxis (PPAP).

### Benchmarking: meta-regression

Five meta-regression models of mortality proportions versus year of study publication were developed using meta-regression methods using DerSimonian and Laird random effects methods using the meta-analysis suite of commands in Stata [29]. Five regression models containing various factors were evaluated. All factors were entered into the meta-regression models without any pre-selection step. Data for the groups as randomly assigned, being intention to treat (ITT) data was used wherever possible. The meta-regression was repeated using on treatment (OT) data from eight studies of TAP-based methods where the data conflicts with that in the two Cochrane reviews of this intervention [21, 22].

## Results

There were 389 groups from 210 studies published between 1985 and 2021 of which 207 groups were sourced from one of the 17 systematic reviews (Additional file 1: Tables S1–S3). The non-decontamination-based infection prevention interventions included various methods and types of ventilator bundles, stress ulcer prophylaxis, enteral feeding, tube feeding, tracheal suctioning, endotracheal tube selection, airway humidification, and probiotic use. The decontamination-based infection prevention interventions included oral care using topical chlorhexidine or other anti-septics and decontamination using TAP with or without the additional use of protocolized parenteral antibiotic prophylaxis (PPAP). All study data is presented in Additional file 1: Tables S1–S4 and Additional file 1: Figs S1–S7. The Additional file 1 includes Figures with mortality data traceable back to the original studies.

The systematic review of decontamination interventions using TAP had been updated by a second team of authors with slightly different study data extraction approaches [21, 22]. Key differences in data are identified in Additional file 1: Table S4. Of note, there were fewer patient reported in eight studies where ‘on treatment’ [22] data had been extracted from the original publications, versus ‘intention to treat’ [21] data obtained by personal communications from the study authors, with the later indicating higher mortality proportions in five of eight studies.

### Characteristics of studies

The groups from observational studies had more patients per group, had a higher group-mean age but otherwise had a similar group-mean APACHE II score and group mean length of ICU stay versus the groups from either category of infection prevention (Table 1). The dispersions in group mean age, group mean APACHE II score and group mean length of stay were similar across all group categories (*p* = NS). A minority of groups included patients from trauma ICU’s or included patients for which < 90% required prolonged MV or reported only late mortality.

**Table 1:** Characteristics of studies

Characteristics	Observational	Non- decontamination	Decontamination
Study characteristics			
Listing	Additional file 1: Table S1	Additional file 1: Table S2	Additional file 1: Table S3
Number of studies^a^	63	90	57
MV for > 48 h for < 90%^b^	9	6	8
PPAP for control groups^c^	0	0	5
Trauma ICUs^d^	5	12	10
Late mortality census^e^	27	22	11
North American ICU	27	36	8
Study publication year (range)	1988–2021	1987–2021	1988–2021
Group characteristics			
Numbers of patients per study group; median (IQR)^f^	296 103–759	69 41–140	75 32–117
Mean patient LOS per study group; mean 95% CI^g^	12.2 10.8– 13.4	11.7 10.3–13.2	14.0 12.1–16.0
Mean patient age per study group; mean 95% CI^h,i^	58.2 56.4–60.1	55 52.8–56.4	52.2 49.6—55.3
Mean patient APACHE II per study group; mean 95% CI^j^	18.9 18.3–19.9	19.6 18.8–20.4	18.7 17.5–19.9
ICU Mortality incidence per 100 patients (mean 95% CI, *n*)^k^			
Observational	22.0 19.5–24.8 (41)		
Concurrent control groups		22.4 20.5–24.4 (84)	27.0^l^^,m,n,o^ 22.9–31.5 (34)
Intervention groups		22.6 20.9–24.5 (80)	23.6^p,q,r^ 21.1–26.4 (56)

^a^Note, 39 studies had more than one observational, control or intervention group and four studies provided both concurrent control and non-concurrent control groups. Hence, the number of groups does not equal the number of studies. Any non-concurrent control groups are included as observational groups in the analysis, except where otherwise stated

^b^Studies for which less than 90% of patients were reported to receive > 48 h of MV

^c^PPAP was used within five control and 36 intervention groups

^d^Trauma ICU arbitrarily defined as an ICU with more than 50% of admissions for trauma

^e^Late mortality is either hospital or beyond day 21 mortality census versus ICU mortality census

^f^Data are median and inter-quartile range (IQR)

^g^Length of stay was similar for the three categories of groups; (*p* = 0.16; one-way ANOVA)

^h^Group mean age was not available for 12 studies

^i^The group mean age for the groups from non-decontamination and decontamination studies were similar but differed from the groups from the observational studies (*p* = 0.002; one-way ANOVA)

^j^The group mean APACHE II score for the groups from non- decontamination, decontamination and the observational studies were similar (*p* = 0.40; one-way ANOVA)

^k^Bartlett’s test for equality of variances, (Chi-square, *df* 4; 37.14) *p* = 0.001

^l^ICU Mortality incidence-non-concurrent control groups of eight studies as in Table S3; 23.2; 18.5–28.7 (9)

^m^ICU Mortality incidence-including on treatment data for control groups of eight studies as in Table S4; 26.5; 22.5–31.0 (34)

^n^ICU Mortality incidence-control groups of studies of studies of antiseptic interventions; 25.7; 18.1–35.1 (9)

^o^ICU Mortality incidence-control groups of studies of topical antibiotic interventions; 27.5; 22.8–32.8 (24)

^p^ICU Mortality incidence–including on treatment data for intervention groups eight studies as in Table S4; 23.3; 20.8–25.9 (56)

^q^ICU Mortality incidence-antiseptic intervention groups; 25.5; 21.0–30.7 (18)

^r^ICU Mortality incidence-topical antibiotic intervention groups; 22.7; 19.8–26.0 (37)

The ICU mortality was similar among the observational groups and the control and intervention groups with the exception of one category. The ICU mortality among the concurrent control groups of decontamination studies were five (5) percentage points higher versus the benchmark (observational groups) and showed greater dispersion. The wider dispersion is reflected as a 95% confidence interval which is approximately four points wider versus that for the other four categories (*p* = 0.001; Bartlett’s test). Late mortality was available for a minority of groups (Additional file 1: Fig. S3).

Among the decontamination studies, ICU mortality among the corresponding control and intervention groups of anti-septic-based and TAP-based decontamination methods were similar.

**Fig. 2:**
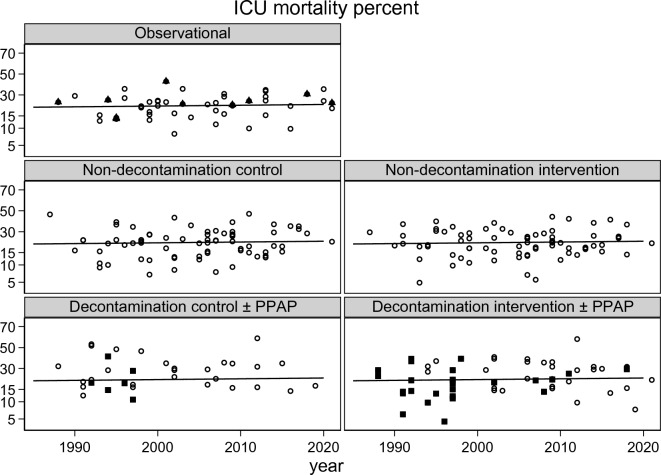
Scatter plot and linear regression of ICU mortality [mortality censured at day 21 or less] incidence versus year of study publication for observational groups and control and interventions groups from studies of non-decontamination and decontamination interventions. The linear regression line in each plot, derived using the observational groups, increased non-significantly versus year of study publication (slope is + 1.2 percentage points per decade; 95% confidence interval − 1.9 to + 4.3; *p* = 0.64) and serves as a benchmark for all plots [symbols; filled triangle = non-concurrent control groups; filled square = groups receiving PPAP; open circle = all other groups; NCC groups appear as observational groups]. PPAP is protocolized parenteral antibiotic prophylaxis. The equivalent plot for late mortality is shown as Additional file 1: Fig S3 and plots with the originating studies indicated in Additional file 1: Figs. S2, S3. Note the *y*-axis is a logit scale

**Fig. 3:**
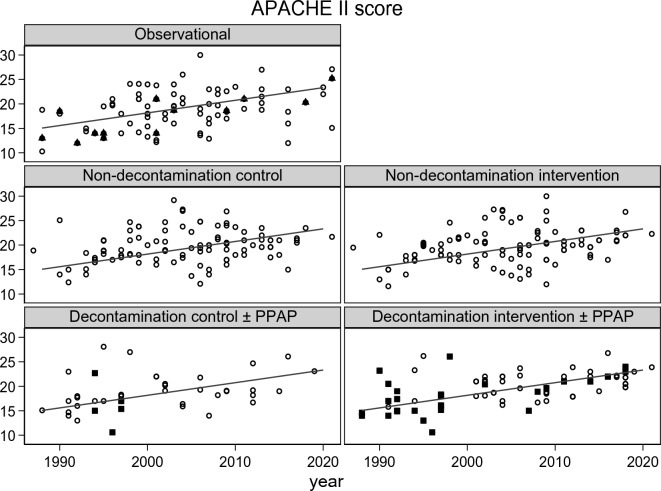
Scatter plot and linear regression of group-mean APACHE II score versus year of study publication for observational groups and control and interventions groups from studies of non-decontamination and decontamination interventions. The linear regression line of APACHE II score versus publication year, derived using the observational groups, increases by + 1.8 points per decade; 95% confidence interval + 0.51 to + 3.2; *p* = 0.007) and serves as a benchmark for all plots [symbols; filled triangle = non-concurrent control groups; filled square = groups receiving PPAP; open circle = all other groups; NCC groups appear as observational groups].The same plot showing each individual group traceable to the original study is shown in Additional file 1: Fig. S4

**Fig. 4:**
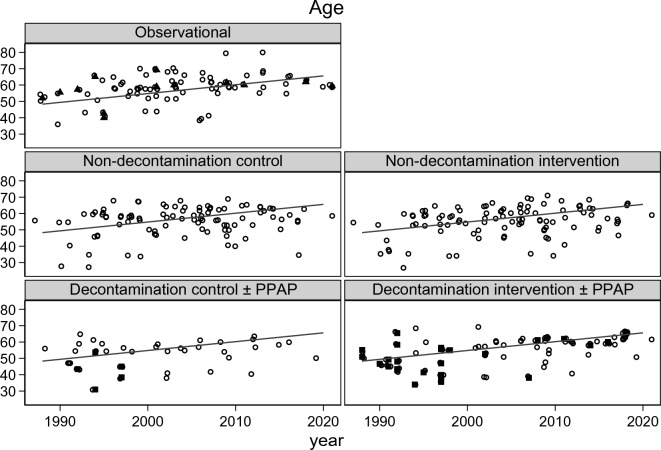
Scatter plot and linear regression of group mean age versus year of publication for groups from studies of infection prevention interventions. The linear regression line in each plot, derived using the observational groups, increased versus year of study publication (slope is + 5.4 years per decade; 95% confidence interval + 2.7 to + 7.9; p = 0.001) and serves as a benchmark for all plots [symbols; filled triangle = non-concurrent control groups; filled square  = groups receiving PPAP; open circle = all other groups; NCC groups appear as observational groups]. The same plot showing each individual group traceable to the original study is shown in Additional file 1: Fig. S5

The ICU mortality (Fig. 2; Additional file 1: Figs. S1, S2) and late mortality (Additional file 1: Fig. S3) did not significantly vary with year of publication. The group-mean APACHE II score (Fig. 3; Additional file 1: Fig. S4) and group-mean age (Fig 4: Additional file 1: Fig. S5) both increased with year of publication and this increase was similar among all study categories. The mortality trend summarized as a LOWESS plot was similar to a summary as a linear regression versus year of study publication (Additional file 1: Fig S1).

### Regression models

The decline in mortality versus year of publication attained statistical significance only within the full meta-regression model (model five), which included adjustment for both group-mean age and group-mean APACHE II score (Table 2). Otherwise, the five meta-regression models gave similar findings with the exception of membership of a trauma ICU, which became non-significant in the models that also included group-mean age. Repeating the analysis with on-treatment data from eight studies of TAP-based methods where the data conflicts with intention to treat data (Additional file 1: Table S4) gave similar findings (data not shown).

**Table 2:** Meta-regression models of mortality incidence^a,b,c,d^

	Model 1	Model 2	Model 3	Model 4	Model 5		
Factor	Coefficient	Coef.	Coef.	Coef.	Coef.	95% CI	*p*
Groups from observational studies (reference group)	− 1.28***	− 1.30***	− 1.71***	− 1.89***	− 2·02**	− 2·4 to − 1·6	0·001
Non-decontamination studies							
● Control groups	− 0·07	− 0·07	− 0·09	− 0·06	− 0·07	− 0·22 to + 0·07	0.34
● Intervention groups	− 0·11	− 0·12	− 0·13	− 0·09	− 0·11	− 0·25 to + 0·03	0.14
Decontamination studies							
● Concurrent control groups	+ 0·19*	+ 0·24**	+ 0·23**	+ 0·26**	+ 0·26**	+ 0·11 to + 0·50	0·009
● Intervention groups	− 0·04	+ 0·06	+ 0·05	+ 0·08	+ 0·07	− 0·11 to + 0·24	0·45
PPAP use^e^		− 0·22	− 0·20	− 0·19	− 0·18	− 0·41 to + 0·05	0.13
MV(per point)^f^		+ 0·003**	+ 0·003**	+ 0·003**	+ 0·003***	+ 0·001 to + 0·005	0.003
Trauma ICU^g^		− 0·36***	− 0·29**	− 0·17	− 0·15	− 0·36 to + 0·06	0.15
Mortality census (late versus ICU)^h^	+ 0·50***	+ 0·47***	+ 0·46***	+ 0·44***	+ 0·44***	+ 0·33 to + 0·55	0.001
APACHE II score (per point)^i^			+ 0.03***		+ 0·02**	+ 0·01 to + 0·04	0.003
Age (per decade)^j^				+ 0.11**	+ 0·09*	+ 0·01 to + 0·17	0.025
Year of publication (per decade)^k^	+ 0·01	− 0·01	− 0·01	− 0·01	− 0·01*	− 0·013 to − 0·001	0.038
Origin from systematic review^l^	+ 0.07	+ 0.06	+ 0.06	+ 0.06	+ 0.08	− 0·02 to − 0·01	0.14

*ICU* intensive care unit, *MV > 90* more than 90% of patients received mechanical ventilation, *PPAP* protocolized parenteral antibiotic prophylaxis

^a^Meta-regression models four and five based on 397 groups (as group mean age is missing for 16 groups), Meta-regression one to three models include all 413 groups

^b^Interpretation. For each model the reference group is the observational study (benchmark) groups and this coefficient equals the difference in logits from 0 (a logit equal to 0 equates to a proportion of 50%; a logit equal to -2.2 equates to a proportion of 10%; a logit equal to -4.6 equates to a proportion of 1%) and the other coefficients represent the difference in logits for groups positive for that factor versus the reference group

^c^*p < 0.05; **p < 0.01; ***p < 0.001

^d^PPAP is the coefficient for those control or intervention groups receiving protocolized parenteral antibiotic prophylaxis

^e^The coefficient representing the increment per percentage point in group level of mechanical ventilation use above 50%

^f^The coefficient representing the increment for admission to a trauma ICU

^g^Late mortality versus ICU mortality [censored at day 21 or before]

^h^Group mean APACHE II score with the coefficient representing the increment for each one-point increase

^i^Group mean age with the coefficient representing the increment for a ten-year increase

^j^Year of study publication with the coefficient representing the increment for each decade post 1980

^k^Increment for a study abstracted in a systematic review

**Fig. 5:**
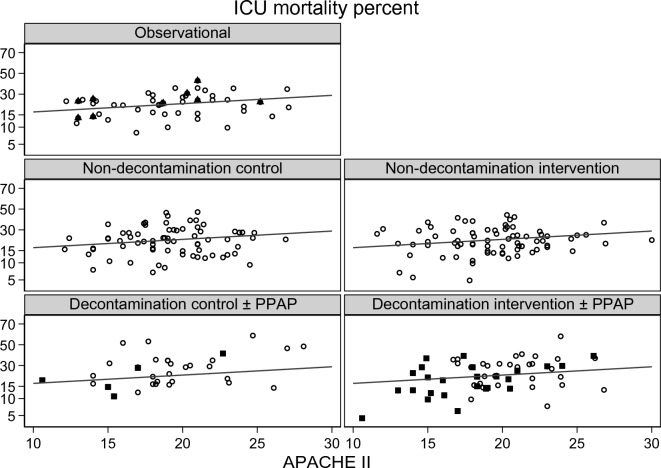
Scatter plot and linear regression of ICU mortality [mortality censured at day 21 or less] incidence versus group mean APACHE II score for groups from studies of infection prevention interventions. The linear regression line in each plot, derived using the observational groups, increased versus year of study publication (slope is + 0.7 percentage points per APACHE II score point; 95% confidence interval + 0.2 to + 1.2; *p* = 0.003) and serves as a benchmark for all plots. Note the *y*-axis is a logit scale [symbols; filled triangle = non-concurrent control groups; filled square = groups receiving PPAP; open circle = all other groups; NCC groups appear as observational groups]. The same plot showing each individual group traceable to the original study is shown in Additional file 1: Fig. S6. An equivalent plot showing the trend for late mortality versus group mean APACHE II score is shown in the Additional file 1: Fig. S7

There is an asymmetrical distribution of ICU (Fig. 2; Additional file 1: Fig. S2) and late (Additional file 1: Fig. S3) mortality incidences for the concurrent control groups from decontamination interventions which are shifted upward and show greater dispersion in relation to the regression line derived using the observational study groups. The higher mortality among the decontamination study control groups is likewise apparent in a scatter plot versus APACHE II score (Fig. 5; Additional file 1: Fig. S4). The higher mortality incidence for concurrent control groups from decontamination interventions was apparent in all five models (Table 2).

## Discussion

Among 210 infection prevention studies published over three and a half decades, there is insignificant variation in mortality risk although the underlying risk, as measured by the group-mean APACHE II score, and group-mean age of the populations have both increased. Only after adjustment for both group-mean age and group-mean APACHE II score is any decrease in mortality over time apparent as a significant trend. Moreover, among broad categories of control, intervention and observational groups, the trends in mortality incidences are similar, except for control groups within studies of decontamination interventions which paradoxically, show higher than expected mortality (Figs. 2 & 5; Additional file 1: Figs. S2, S3, S6 & S7).

A premise underlying the research questions and the analysis here is that acquired infections in the ICU is a key and potentially preventable driver of mortality in the ICU. Interventions, such as decontamination-based methods, that consistently prevent acquired infection, should impact the mortality trend versus interventions, such as non-decontamination-based methods, that offer less infection prevention [30–32]. Hence, an analysis that incorporates studies of infection prevention interventions having different efficacy will provide triangulation to the analysis of mortality trends.

The findings here differ to trends derived based on analyses of either administrative databases [33, 34]. or based on control arms from RCT’s of either sepsis treatments or lung protective ventilation strategies [35–40]. These analyses have generally shown declining mortality, either absolute or risk adjusted, by as much as 10 percentage points per decade over observation periods of up to two decades.

Each of these types of prior analyses had acknowledged limitations threatening their validity [41–43]. For analyses based on administrative databases, changes in admission, discharge and coding practices over time together with the uncertain generalizability to other jurisdictions threaten their validity.

For analyses based on published studies, these are based on a limited number of observations, typically with less than 70 studies. Several are adjusted using other types of severity score measure, for example SOFA or SAPS 2, in the analyses, which further dilutes the study power. In some cases, these measures show increased and in other cases, decreased illness severity over time. Changes in disease definitions and variable patient recruitment success also threaten their validity. Moreover, with restriction to specific patient groups of interest, such as studies of ventilation strategies, the overall mortality and length of stay are generally > 30% and > 10 days, respectively, with a limited range among the included studies. By contrast, with all ICU admissions included, as in database analyses, the overall mortality and length of stay are generally < 20% and < 5 days, respectively. Hence, the ICU populations in prior analyses may be either too broad and overall too low risk, or too specific and overall too high risk, respectively, to be able to adequately address the question of whether underlying risk using the APACHE II score is relevant to evaluating the mortality trends over time.

By contrast, the 210 studies here were included based on being either observational or having evaluated prevention strategies potentially applicable to MV patients over more than three decades. The search strategy enables the providence of the data to be sourced to individual studies within individual systematic reviews which form the evidence base for these infection prevention interventions. Of note, studies limited to specific patient groups such as those with ARDS or those post cardiac surgery were deliberately excluded as these populations would likely be at too high and too low risk, respectively.

A feature of this analysis is the broad range in each the following; mortality incidences, mortality census, group mean APACHE II severity scores, group mean age and group mean length of stay, among the included studies. This breadth is a strength of the current analysis towards addressing the research questions. Additional strengths are that ICU mortality would likely be completely ascertained for all randomized patients within RCCT’s at end points defined in time (ICU versus late) [43]. There is evidence of incomplete follow up for > 8 studies of TAP, although this has minimal impact on the analysis. Given the breadth in study interventions, it is paradoxical that the dispersion in mortality incidence is greatest among the control groups of the decontamination studies whereas there is no corresponding dispersion in the various metrics of underlying risk.

Regarding the first research question, it is reasonable to presume from previous analyses of both databases and literature studies that mortality has improved over time [33–40]. However, the analysis of the infection prevention studies here indicates that the APACHE II score may not adequately account for the increasing risk associated with increasing age of ICU populations over three decades. This is surprising given that the APACHE II score better predicts ICU mortality in the elderly than does age, whereas age better predicts long-term mortality than does the APACHE II or SAPS2 score [44, 45].

Regarding the second research question, the mortality incidences for decontamination and non-decontamination intervention groups were broadly similar to benchmark. The use of topical antiseptics is of interest given the recent concern that they may be associated with increased mortality risk despite reduction in VAP incidence [46]. However, the mortality incidence for the intervention groups receiving anti-septics were similar to those receiving TAP and show no significant increase in comparison to the benchmark. In striking contrast, the higher mortality among the control groups of decontamination studies versus the benchmark is evident in all models with or without adjustment for underlying risk.

There are four limitations to this analysis. First, it is not a systematic review of the interventions. Rather, it is an opportunistic analysis of available data drawn mostly from systematic reviews which form the evidence base versus the broader literature. Second, the analysis of trend in mortality is adjusted using group level indicators of risk. Without patient level data, the trends in individual level risk, or risk within specific sub-groups of interest, such as those with immunosuppression or different categories of ICU admission or different types of acquired infection [47], were not able to be analyzed. Of note in this regard, contextual effects are observable only at the group level and only by reference to an external benchmark [3, 4].

Third, diagnosis related categories are integral in the accurate estimation of underlying patient risk and these were not included in the analysis with the exception of trauma ICU admission as a group level risk factor as an ecological type analysis. Of note, group-mean age confounds trauma ICU admission as factors in the analysis.

Fourth, the analysis is exploratory as only a limited number of factors were entered into the meta-regression models and no interaction or non-linear effects were explored. The year of study publication approximates the date that the patients were enrolled in each study. Studies reporting a severity score other than APACHE II score, such as SAPS2, were not included here but similar findings were noted previously in an earlier meta-analysis for which only group-mean age was available as a proxy for underlying mortality risk [28]. Of the 210 studies, only 126 are common to both analyses.

Finally, the true extent of publication bias among studies of prevention interventions is difficult to estimate. It has been noted that among studies of various treatments for sepsis and studies of different ventilation strategies, that in comparing studies with positive versus negative outcomes, the former had higher control group mortality incidences whereas the intervention group incidences are similar among studies regardless of negative or positive outcomes [39, 40]. Publication bias is thought to contribute to this phenomenon but this would be expected to be similar for both studies of non-decontamination-based methods as for decontamination-based methods. Here, there is evidence for higher control group mortality despite comparable group level measures of underlying risk in these decontamination studies.

There is no reason to expect publication bias to differentially impact the findings of systematic reviews within the Cochrane review database of studies of decontamination versus non-decontamination studies.

Moreover, four observations suggest that contextual effects resulting from the decontamination interventions underlie the disparities in the mortality incidence amongst the concurrent control groups of these studies.

First, despite the higher and more disperse ICU mortality incidences among the control groups of decontamination studies, the measures of, and dispersion amongst, group mean age, group mean length of stay and group mean APACHE II score were similar cross all categories of study.

Second, the studies of TAP with a non-concurrent control design generally show less infection and mortality prevention and lack the disparities in the control group mortality incidences [48]. Likewise, studies of TAP that had concurrent control groups receiving PPAP had control group mortality incidences more similar to benchmark.

Two recent cluster randomized trials using evidence-based infection prevention interventions including TAP, one French [49] and one Australian [9], have not been able to recapitulate the mortality reduction apparent within the systematic reviews of RCCT’s of these interventions.

Third, the higher mortality incidences noted here accord with other paradoxical observations in relation to higher incidences of bacteremia, candidemia and VAP among concurrent control groups of studies using TAP to achieve decontamination as infection prevention [3]. These observations, whilst paradoxical, remain consistent with the effect of TAP being mediated by a control, or lack thereof, of gut overgrowth (COGO) amongst both concurrent control and intervention groups [50, 51, 52].

Resolving the paradox between the infection prevention and mortality prevention that is seen in RCCT’s of TAP but not in CRT’s of TAP would require a purpose designed study to estimate the contextual effect arising from the use of decontamination interventions within the ICU. Such a trial would be both ethically and logistically problematic [53].

## Conclusion

The incidence of ICU mortality among groups of studies of infection prevention interventions has changed insignificantly over three decades whilst underlying severity, as reflected in the group-mean APACHE II score and group-mean age have both increased. The paradoxical higher mortality within concurrent control groups of decontamination RCCT’s remains unexplained.

## Take-home message

Has either the underlying disease severity or the mortality incidence among ICU patients receiving MV in the literature changed in recent decades and what is the impact of infection prevention interventions?

Among studies of infection prevention interventions over three decades, the mortality incidence has remained constant whilst the underlying disease severity, as APACHE II score, and age have increased.

A decrease in mortality over the past three decades becomes apparent only after adjusting for both underlying disease severity and age.

## Supplementary Information

**Additional file 1. Table S1**: Mortality data: observational studies. **Table S2**: Mortality data: non-decontamination methods of VAP prevention. **Table S3**: Mortality data: decontamination methods of infection prevention. **Table S4**: On treatment [OT] versus intention to treat [ITT] mortality data discrepancies. **Figure S1**. LOESS plot of ICU mortality versus year. **Figure S2**. ICU mortality versus year of publication. **Figure S3**. Late mortality versus year of publication. **Figure S4**. Group mean APACHE II score versus year of publication. **Figure S5**. Group mean age versus year of publication. **Figure S6**. ICU mortality versus group mean APACHE II score. **Figure S7**. Late mortality versus group mean APACHE II score.

## Data Availability

All data generated or analysed during this study are included in this published article [and its Additional files].

## Abbreviations

ICU: Intensive care unit
MV: Mechanical ventilation
CRT: Cluster randomized trials
RCCT: Randomized concurrent controlled trial
SDD: Selective digestive decontamination
TAP: Topical antibiotic prophylaxis
PPAP: Protocolized parenteral antibiotic prophylaxis
COGO: Control of gut overgrowth
