# Supplementary material for: Trends in ICU mortality and underlying risk over three decades among mechanically ventilated patients. A group level analysis of cohorts from infection prevention studies
Source: Ann Intensive Care. 2023 Jul 11;13:62. doi: 10.1186/s13613-023-01159-0 (PMC10335996; doi:10.1186/s13613-023-01159-0)
Supplement: Supplementary file 1 — Additional file 1. Table S1: Mortality data: observational studies. Table S2: Mortality data: non-decontamination methods of VAP prevention. Table S3: Mortality data: decontamination methods of infection prevention. Table S4: On treatment [OT] versus intention to treat [ITT] mortality data discrepancies. Figure S1. LOESS plot of ICU mortality versus year. Figure S2. ICU mortality versus year of publication. Figure S3. Late mortality versus year of publication. Figure S4. Group mean APACHE II score versus year of publication. Figure S5. Group mean age versus year of publication. Figure S6. ICU mortality versus group mean APACHE II score. Figure S7. Late mortality versus group mean APACHE II score. [file 13613_2023_1159_MOESM1_ESM.pdf]

Trends in ICU mortality and underlying risk among 210 infection prevention studies

Online Data Supplement

James C Hurley

Supplemental file contents:

Table S1: Mortality data: observational studies 2-3

Table S2: Mortality data: non-decontamination methods of VAP prevention 4-9

Table S3: Mortality data: decontamination methods of infection prevention 10-15

Table S4: On treatment [OT] versus intention to treat [ITT] mortality data discrepancies 15

References 16–26

Figure S1. LOESS plot of ICU mortality versus year 26

Figure S2. ICU mortality versus year of publication 27

Figure S3. Late mortality versus year of publication 28

Figure S4. Group mean APACHE II score versus year of publication 29

Figure S5. Group mean age versus year of publication 30

Figure S6. ICU mortality versus group mean APACHE II score 31

Figure S7. Late mortality versus group mean APACHE II score 32

Table S1: Mortality data: observational studies [a]

Author	Year	Ref	Notes	Age [a]	APACHE II	Census	Mortality (n)	Patients (n)	Mortality (%)
Akça	2000	1		44	13		66	260	25
Apostolopoulou	2003	2		52	20		62	175	35
Artinian_all	2006	3		62	21		781	4049	19
Bonten	1996	4		58	21		50	141	35
Boots	2008	5		56	20		68	412	17
Cavalcanti	2006	6	T		14		14	62	23
Cenderero	1999	7		59	14		30	123	24
Chevret	1993	8	<90		14		158	996	16
Cook	1998	9		55	24		207	1014	20
Craven-medical	1988	10		54	19	L	231	526	44
Craven-surgical	1988	10		51	10	L	184	799	23
Eddleston	1994	11		50	14	L	10	26	38
Ensminger	2006	12		67	30	L	37	92	40
Ertugrul	2006	13	T	40	14	L	29	100	29
Fagon	1996	14	<90	58	20		542	1978	27
Fieselmann	1999	15		70	22	L	81	224	36
Francois	2021	16		61	24	L	76	194	39
Hart_Scottish	2020	17		60	22		57	227	25
Hebert -liberal	1999	18	<90	58	21		68	420	16
Hebert -restr	1999	18	<90	57	21		56	418	13
Heyland	1999	19		56	24		113	639	18
Hyllienmark	2007	20		58	16	L	59	329	18
Ibrahim'00	2000	21		57	17	L	589	3668	16
Ibrahim'00	2000	22	<90	58	24	L	189	492	38
Ibrahim'01	2001	23	<90	69	24	L	301	880	34
Jacobs	1990	24	T	36	18		8	24	33
Jaimes	2007	25		41	17		50	270	19
Kasuya	2011	26		66	19		11	111	10
Kirschenbaum	2002	27		68	17		3	37	8
Ko	2013	28		80	19		230	1453	16
Kollef' 93	1993	29		44	15		36	277	13
Kollef '95	1995	30		63	20	L	119	314	38
Kollef '97	1997	31		61	18	L	131	521	25
Laupland	2004	32	<90	65	26		657	4473	15
León	2009	33		60	18		240	1107	22
León	2016	34	<90	67	18		50	233	21
Luna	2003	35		67	20	L	32	63	51
Luna	2006	36		63	19	L	40	76	53
Magnason	2008	37		58	18		86	278	31
Muscedere	2013	38		60	23		335	1320	25
Nguyen	2008	39			22		8	28	29
Osmon	2003	40	<90	58	22	L	248	893	28

Author	Year	Ref	Notes [b]	Age [c]	APACHE II	Census [a]	Mortality (n)	Patients (n)	Mortality (%)
Petri	1997	41			14	L	133	409	33
Ramirez	2016	42		61	23		43	440	10
Rello'03	2003	43		62	16	L	34	110	31
Rodrigues	2009	44		79	17	L	143	233	61
Ruiz	2000	45		66	20	L	31	76	41
Schweickert	2004	46		61	21	L	28	60	47
Schweickert	2004	46		56	20	L	24	66	36
Shahin	2013	47		55	27		92	267	34
Sinuff	2010	48		59	24	L	100	330	30
Sinuff	2009	48		58	23	L	98	330	30
Sinuff	2007	48		62	23	L	126	330	38
Sinuff	2008	48		60	23	L	119	330	36
Sofianou	2000	49			18		49	198	25
Steen	2021	50		60	27		542	2720	20
Suka	2007	51		63	13		1022	8892	11
Tan	2016	52		54	12	L	128	354	36
Tan	2016	52		64	16	L	112	264	42
Tejada-Artigas	2001	53	T	45	12		25	103	24
Timsit	1996	54		65	20	L	168	387	43
Urli	2002	55		51	18		30	178	17
van der Kooi	2007	56		61	20		360	1516	24
Violan	1998	57		53	16		67	314	21
Violan	2000	58		53	15		19	88	22
Walsh	2013	59		68	22		17	51	33
Walsh	2013	59		67	20		14	49	29
Warren	2003	60		69	24		301	819	37
Woske	2001	61		53	13	L	36	103	35
Xie	2020	62	<90		23		2983	8474	35
Zygun	2006	63	T	38	18	L	38	134	28

Table S1 Footnotes

a. Studies [n=22] derived from the following systematic reviews [210-212].

b. Notes; T – Data originating from a study for which the majority of ICU admission were for trauma; <90 – less than 90% of patients received > 24 hours of MV

c. Age – Group mean (or median) age (years)

d. Census - L – Late mortality being day 28 or hospital mortality

Table S2: Mortality data: non-decontamination methods of infection prevention [a]

Author	Year	Ref	Notes [b]	Age [c]	APACHE II	Census [d]	Mortality (n)	Patients (n)	Mortality (%)
**control groups**									
Bloos	2009	64		63	24		37	133	28
Crunden	2005	65			19		52	246	21
DeLuca	2017	66	T	35	21		63	195	32
Hawe	2009	67			19		112	374	30
Morris	2011	68		60	21		367	1460	25
Parisi	2016	69		59	15		79	226	35
Acosta-escribano	2010	70	T	41	18		9	54	17
Altintas	2011	71		58	23	L	20	41	49
Arabi	2015	72		51	21		85	446	19
Ben-Menachem	1994	73	<90	60	17		5	50	10
Ben-Menachem	1994	73	<90	60	17	L	10	50	20
Boivin	2001	74		48	16		7	39	18
Bonten '95	1995	75		57	21		26	67	39
Bonten	1995	75		57	21	L	32	67	48
Bonten '96	1996	76		68	17		6	30	20
Conrad	2005	77		57	23		22	181	12
Cook	1998	78		59	25		140	596	23
Davies	2002	79		54	21		5	39	13
Davies	2012	80		54	20		12	89	13
Driks	1987	81		55	19		32	69	46
Eddleston '91	1991	82		54	12		7	30	23
Esparza	2001	83		50	17	L	11	27	41
Fabian	1993	84	T	35	14		16	99	16
Grau	2011	85	<90	65	18		13	68	19
Grau-Carmona	2011	86		65	19		11	71	15
Hanisch	1998	87		58	18		6	28	21
Hanisch	1998	87		55	21		7	57	12
Harvey	2014	88	<90	63	20		352	1197	29
Harvey	2014	88	<90	63	20	L	393	1188	33
Heidegger	2013	89		61	22		20	153	13
Heyland	1999	90		55	22		7	59	12
Heyland	2013	91		61	21		42	267	16
Heyland	2013	91		64	24		35	252	14
Hsu	2009	92		68	21		24	62	39
Ibrahim '02	2002	93		57	25	L	15	75	20
Johnstone	2021	94		60	22		296	1332	22
Kantorova	2004	95		46	18	L	4	25	16
Kearns_C	2000	96		49	20	L	6	23	26
Knight	2009	98		50	17		34	129	26

Table S2 (continued): Mortality data: non-decontamination methods of infection prevention [a]

Author	Year	Ref	Notes [b]	Age [c]	APACHE II	Census [d]	Mortality (n)	Patients (n)	Mortality (%)
**control groups**									
Knight	2009	98		50	17	L	42	129	33
Kortbeek	1999	99	T	35	18		3	43	7
Kotzampassi	2006	100	T	56	19		9	30	30
Levy	1997	101	<90	57	18		12	35	34
Martin	1993	102		60	15		7	66	11
Montecalvo_G	1992	103		45	17		5	19	26
Montejo	2002	104	<90	59	19		22	51	43
Morrow	2010	105		53	24		12	68	18
Muscedere	2018	106		63	24		30	105	29
Nguyen	2008	107		55	21		4	14	29
Nguyen	2008	107		56	24	L	6	14	43
O'Keefe	1998	108	T	34	23		11	49	22
Pickworth	1993	109	T	27	18		4	44	9
Prod'hom	1994	110		46	17	L	25	83	30
Prod'hom_A	1994	110		46	17		17	81	21
Prod'hom	1994	110		46	17	L	32	81	40
Rice	2009	111		53	27		22	98	22
Ruiz-Santana	1991	112		37	15		5	19	26
Ruiz-Santana	1991	112		39	15		7	30	23
Spindler-Vesel	2007	113	T	35	14		5	87	6
Terzi	2009	114		45	16		3	11	27
Thomason	1996	115		28	19		22	140	16
White	2009	116		54	25		5	54	9
Wischmeyer	2016	117		55	21	L	8	52	15
Ahrens	2004	118		63	27	L	58	137	42
Barquist	2006	119	T	50	12		5	31	16
Boots '97	1997	120		53	19		7	33	21
Boots'06	2006	121		58	20		29	190	15
Darvas	2003	122		64	29	L	17	53	32
David	2011	123		44	21		47	100	47
Deppe	1990	124		53	25		11	38	29
Drakulovic	1999	125		67	24	L	13	47	28
Fink	1990	126	T	29	14		8	48	17
Keeley	2007	127		64	20		4	13	31
Keeley	2007	127		64	20	L	4	13	31
Kollef '95	1995	128		59	19		36	153	24
Kollef '97	1997	129		57	18	L	64	263	24
Kollef '98	1998	130		59	18	L	39	147	27
Kollef'08	2008	131		62	22	L	198	743	27
Leaf	2014	132	<90	58	21		6	31	19

Table S2 (continued): Mortality data: non-decontamination methods of infection prevention [a]

Author	Year	Ref	Notes [b]	Age [c]	APACHE II	Census [d]	Mortality (n)	Patients (n)	Mortality (%)
**control groups**									
Lorente '03	2003	133		58	17		28	116	24
Lorente '04	2004	134		56	17		51	143	36
Lorente'05	2006	135		58	16		50	233	21
Lorente'06a	2005	136		58	14		30	221	14
Lorente'06b	2006	137		55	19		12	51	24
Lorente'07	2007	138		60	15		32	140	23
Lorente'14	2014	139		63	18		55	150	37
MacIntyre	1999	140		56	16		14	51	27
Mahmoodpoor	2017	141		55	21		48	138	35
Memish	2001	142		46	21		30	120	25
Nair	2015	143		56	18		4	25	16
Ntoumenopoulos	2002	144		65	19		3	36	8
Patman	2009	145	T	41	20	L	21	72	29
Rumbak	2004	146		63	27	L	37	60	62
Smulders	2002	147		63	23		10	75	13
Topeli	2004	148		68	24	L	25	37	68
Traver	1995	149	T	59	18	L	19	59	32
Valencia	2007	150		64	19		16	69	23
Valles	1995	151		63	19		28	77	36
van Nieuwenhoven	2006	152		63	25		30	109	28
van Nieuwenhoven	2006	152		63	25	L	38	109	35
Young	2013	153		64	20	L	139	451	31

Table S2 (continued): Mortality data: non-decontamination methods of infection prevention [a]

Author	Year	Ref	Notes [b]	Age [c]	APACHE II	Census [d]	Mortality (n)	Patients (n)	Mortality (%)
**Intervention groups**									
Bloos	2009	64	I	66	25		36	141	26
Crunden	2005	65	I		17		64	334	19
DeLuca	2017	66	I	55	23		38	153	25
DeLuca	2017	66		47	21		49	192	26
Hawe	2009	67	I		20		49	215	23
Morris	2011	68	I	59	22		101	501	20
Parisi	2016	69	I	58	17		56	136	41
Acosta-escribano	2010	70	T	35	16		6	50	12
Altintas	2011	71		58	20	L	13	30	43
Arabi	2015	72		50	21		72	448	16
Ben-Menachem	1994	73	<90	59	18	L	28	100	28
Ben-Menachem	1994	73	<90	59	18		19	100	19
Boivin	2001	74		49	17		7	39	18
Bonten '95	1995	75		59	20		24	74	32
Bonten	1995	75		59	20	L	27	74	36
Bonten '96_I	1996	76		65	19		9	30	30
Conrad	2005	77		55	25		27	178	15
Cook	1998	78		59	25		138	604	23
Davies	2002	79		56	20		4	34	12
Davies	2012	80		51	20		13	91	14
Driks	1987	81		54	20		18	61	30
Eddleston '91	1991	82		44	12		8	30	27
Esparza	2001	83		45	16	L	10	27	37
Fabian	1993	84	T	35	14		34	179	19
Grau	2011	85	<90	68	19		9	59	15
Grau-Carmona	2011	86		62	19		11	61	18
Hanisch	1998	87		53	18		12	44	27
Harvey	2014	88	<90	63	20	L	409	1195	34
Harvey	2014	88	<90	63	20		317	1191	27
Heidegger	2013	89		60	23		28	152	18
Heyland	1999	90		55	22		15	61	25
Heyland	2013	91		65	23		47	270	17
Heyland	2013	91		63	21		49	270	18
Hsu	2009	92		70	20		26	59	44
Ibrahim '02	2002	93		59	26	L	20	75	27
Johnstone	2021	94		60	22		279	1318	21
Kantorova	2004	95		47	19	L	11	71	15
Kantorova	2004	95		44	18	L	14	72	19
Kantorova	2004	95		51	19	L	13	69	19
Kearns_I	2000	96		54	22	L	5	21	24

Table S2 (continued): Mortality data: non-decontamination methods of infection prevention [a]

Author	Year	Ref	Notes [b]	Age [c]	APACHE II	Census [d]	Mortality (n)	Patients (n)	Mortality (%)
**Intervention groups**									
Klarin [e]	2018	97		66	24	L	12	68	18
Klarin [e]	2018	97		66	22	L	14	69	20
Klarin [e]	2018	97		66	24		11	68	16
Klarin [e]	2018	97		66	22		10	69	14
Knight	2009	98		50	17	L	35	130	27
Knight	2009	98		50	17		28	130	22
Kortbeek	1999	99	T	34	18		4	37	11
Kotzampassi	2006	100	T	53	19		5	35	14
Levy	1997	101	<90	57	20		11	32	34
Martin	1993	102		59	17		8	65	12
Montecalvo	1992	103	T	51	16		5	19	26
Montejo	2002	104	<90	57	18		19	50	38
Morrow	2010	105		55	23		15	70	21
Muscedere	2018	106		66	27		39	107	36
Nguyen	2008	107		55	23	L	6	14	43
Nguyen	2008	107		56	23		4	14	29
O'Keefe	1998	108	T	34	21		6	47	13
Pickworth	1993	109	T	27	18		2	39	5
Prod'hom	1994	110		52	17	L	27	80	34
Prod'hom_R	1994	110		52	17		16	80	20
Rice	2009	111		54	27		20	102	20
Ruiz-Santana	1991	112		39	15		5	24	21
Ruiz-Santana	1991	112		37	15		7	19	37
Ruiz-Santana	1991	112		39	15		7	24	29
Spindler-Vesel	2007	113	T	48	14		2	26	8
Terzi	2009	114		44	12		4	13	31
Thomason	1996	115		33	18		37	148	25
Thomason	1996	115		31	17		15	136	11
White	2009	116		50	30		11	50	22
Wischmeyer	2016	117		55	21	L	17	73	23
Ahrens	2004	118		62	27	L	41	97	42
Barquist	2006	119	T	54	13		2	29	7
Boots'97	1997	120		52	19		6	42	14
Boots '97	1997	120		49	18		4	41	10
Boots'06_SHW	2006	121		55	20		13	94	14
Boots'06_DHW	2006	121		56	19		21	97	22
Darvas	2003	122		66	27	L	15	48	31
David	2011	123		42	21		42	100	42
Deppe	1990	124		53	22	L	12	46	26
Drakulovic	1999	125		63	21		7	39	18

Table S2 (continued): Mortality data: non-decontamination methods of infection prevention [a]

Author	Year	Ref	Notes [b]	Age [c]	APACHE II	Census [d]	Mortality (n)	Patients (n)	Mortality (%)
**Intervention groups**									
Fink	1990	126	T	35	13		10	51	20
Keeley	2007	127		69	20		3	17	18
Keeley	2007	127		69	20	L	4	17	24
Kollef '95	1995	128		61	20		45	147	31
Kollef '97	1997	129		58	18	L	67	258	26
Kollef '98	1998	130		58	17	L	40	163	25
Kollef'08	2008	131		61	21	L	233	766	30
Leaf	2014	132	<90	68	18		7	36	19
Lorente '03	2003	133		57	15		37	114	32
Lorente '04	2004	134		58	14		47	161	29
Lorente'05	2006	135		59	16		52	210	25
Lorente'06a	2005	136		59	14		31	236	13
Lorente'06b	2006	137		56	18		13	53	25
Lorente'07	2007	138		61	15		26	140	19
Lorente'14	2014	139		60	18		51	134	38
MacIntyre	1999	140		56	17		15	52	29
Mahmoodpoor	2017	141		55	23		36	138	26
Memish	2001	142		48	21		40	123	33
Nair	2015	143		48	21		5	25	20
Ntoumenopoulos	2002	144		65	21		6	24	25
Patman	2009	145	T	46	21	L	13	72	18
Rumbak	2004	146		63	27	L	19	60	32
Smulders	2002	147		64	22		12	75	16
Topeli	2004	148		61	26	L	27	41	66
Traver	1995	149	<90, T	53	20	L	12	44	27
Valencia	2007	150		64	18		20	73	27
Valles	1995	151		63	21		30	76	39
van Nieuwenhoven	2006	152		65	26		29	112	26
van Nieuwenhoven	2006	152		65	26	L	39	112	35
Young	2013	153		64	20	L	141	448	31

Table S2 footnotes

a. Studies [n=55] derived from the following systematic reviews [213-222].
b. Notes; T – Data originating from a study for which the majority of ICU admission were for trauma; <90 – less than 90% of patients received > 24 hours of MV
c. Age – Group mean (or median) age (years)
d. Census - L – Late mortality being day 28 or hospital mortality
e. Klarin – the control group received topical chlorhexidine and is included as a second intervention group

Table S3: Mortality data: Decontamination methods of infection prevention [a]

Author	Year	Ref	Notes [b]	Age [c]	APACHE II	Census [d]	Mortality (n)	Patients (n)	Mortality (%)
**control groups**									
Bellissimo-Rodrigues	2009	154	<90	54	19		33	96	34
Chua	2004	156		55	16	L	10	20	50
Dale_CHORAL NC	2021	157		59	25		399	1691	24
Fu	2019	159		50	23		7	40	18
Genuit (WP)	2001	160		69	14	L	7	39	18
Koeman	2006	161		62	22		38	130	29
Kollef'06	2006	162		58	19		63	347	18
Long	2012	163		64	18		5	30	17
Lorente'12	2012	164		60	19		69	219	32
MacNaughton	2004	165		56	16	L	29	93	31
Meinberg	2012	166	T	41	17	L	9	24	38
Özçaka	2012	167		56	25		19	32	59
Scannapieco	2009	169		50	19		8	49	16
Swan	2016	170	<90	60	26		24	164	15
Tantipong	2008	171		60	18		37	105	35
Abele-Horn	1997	172	T	45	18		5	30	17
Aerdts	1991	173	Lib	48	23		12	60	20
Bergmans NC	2001	174		59	21		26	61	43
Bergmans	2001	174		58	22	L	32	78	41
Bergmans	2001	174		58	22		27	78	35
Bergmans	2001	174		59	21	L	27	61	44
Blair	1991	175		47	14		22	130	17
Boland	1991	176	Lib, T		17		4	32	13
Bonten CC	1994	177	<90	62	17		6	21	29
Bonten NC	1994	177		65	14		14	54	26
Cockerill	1992	178		65	18		16	75	21
de Jonge NC	2003	179	<90	60	19	L	146	468	31
de Jonge	2003	179	<90	60	19		107	468	23
de Smet NC	2009	180	<90	61	19		443	1990	22
de Smet NC	2009	180	<90	61	19	L	544	1990	27
de_Latorre	1995	181		54	17	L	31	80	39
Georges	1994	182	PP, T	31	15		5	33	15
Hammond	1992	183	Lib, PP	44	14		31	160	19
Hammond-post NC	1995	184	<90		13		100	719	14
Hammond-pre NC	1995	184	<90		14		59	406	15
Jacobs	1992	185		55	18		23	43	53

Table S3: Mortality data: Decontamination methods of infection prevention (continued) [a]

Author	Year	Ref	Notes [b]	Age [c]	APACHE II	Census [d]	Mortality (n)	Patients (n)	Mortality (%)
**control groups**									
Karvouniaris	2015	186		58	19		29	84	35
Kerver	1988	187		56	15		15	47	32
Krueger	2002	188		54	20		75	262	29
Laggner	1994	189		54	23		14	34	41
Ledingham	1988	190	<90	52	13		39	161	24
Lingnau	1997	191	PP, T	38	15		16	148	11
McClelland NC	1990	192		56	19	L	7	12	58
Palomar Ctx	1997	193	Lib, PP	45	17		14	49	28
Pneumatikos	2002	194	T	37	19		7	30	23
Pugin	1991	195	Lib, T	46	15	L	11	41	27
Rocha	1992	196	Lib, T	44	16		40	77	52
Sanchez-Garcia	1998	197		55	27		65	140	46
Stoutenbeek	1996	198	Lib, PP		11		8	42	20
Stoutenbeek '07	2007	199	T	41	14		44	200	22
Verwaest	1997	200	Lib	56	18		40	220	18
Wiener	1995	201		59	28		15	31	48
Winter CC	1992	202		60	13	L	40	92	43
Winter NC	1992	202		57	12	L	34	84	40
Wittekamp NC	2018	203		62	20 [e]		691	2251	31
Wittekamp NC	2018	203		62	20 [e]	L	701	2198	32
Wood	2002	204	T	41	21		6	20	30

Author	Year	Ref	Notes [b]	Age [c]	APACHE II	Census [d]	Mortality (n)	Patients (n)	Mortality (%)
intervention groups									
Bellissimo-Rodrigues	2009	154	<90	63	17		35	98	36
Bellissimo-Rodrigues	2014	155	<90	60	22		40	127	31
Bellissimo-Rodrigues	2014	155	<90	53	23		37	127	29
Chua	2004	156		51	17	L	12	22	55
Dale_CHORAL	2021	157		60	24		330	1555	21

Table S3: Mortality data: Decontamination methods of infection prevention (continued) [a]

Author	Year	Ref	Notes [b]	Age [c]	APACHE II	Census [d]	Mortality (n)	Patients (n)	Mortality (%)
intervention groups									
de Lacerda Vidal	2017	158		59	22	L	20	105	19
de Lacerda Vidal	2017	158		63	22	L	27	108	25
Fu	2019	159		50	23		3	40	8
Genuit	2001	160		69	18	L	7	56	13
Koeman-Ch	2006	161		61	22		49	127	39
Koeman - ChC	2006	161		62	24		46	128	36
Kollef'06	2006	162		61	20		80	362	22
Long	2012	163		60	18		3	31	10
Lorente'12	2012	164		61	18		62	217	29
MacNaughton	2004	165		58	16	L	29	101	29
Meinberg	2012	166	T	40	18	L	13	28	46
Özçaka	2012	167		61	24		17	29	59
Pobo	2009	168		55	19		23	73	32
Pobo	2009	168		53	19		16	74	22
Scannapieco	2009	169		45	19		8	47	17
Scannapieco	2009	169		48	20		8	50	16
Swan	2016	170	<90	59	27		22	161	14
Tantipong	2008	171		57	17		36	102	35
Abele-Horn	1997	172	T	40	16		11	58	19
Aerdts	1991	173	Lib, T	45	21		4	28	14
Bergmans	2001	174		57	21		25	87	29
Bergmans	2001	174		57	21	L	30	87	34
Blair	1991	175		49	14		17	126	13
Boland	1991	176	Lib, T		17		2	32	6
Bonten	1994	177		67	17		7	22	32
Cockerill	1992	178	<90	66	19		11	75	15
de Jonge	2003	179	<90	60	19		69	466	15
de Jonge	2003	179	<90	60	19	L	113	466	24
de Smet NC SOD	2009	180		61	20	L	502	1904	26
de Smet NC SOD	2009	180		61	20		416	1904	22
de Smet NC SDD	2009	180		62	20	L	546	2045	27
de Smet NC SDD	2009	180		62	20		440	2045	22
Garbino	2002	205		56	21		41	101	41
Garbino_Fl	2002	205		53	21		40	103	39
Garbino'04_TPN	2004	206			22		12	29	41
Garbino'04_EN	2004	206			21		28	71	39
Georges	1994	182	T	34	15		3	31	10

Table S3: Mortality data: Decontamination methods of infection prevention (continued) [a]

Author	Year	Ref	Notes [b]	Age [c]	APACHE II	Census [d]	Mortality (n)	Patients (n)	Mortality (%)
intervention groups									
Hammond	1992	183	Lib	44	14		34	162	21
Hammond	1995	184			13		54	403	13
Jacobs	1992	185		48	17		14	36	39
Karvouniaris	2015	186		60	19		25	84	30
Kerver	1988	187		55	15		14	49	29
Krueger	2002	188		53	20		52	265	20
Laggner	1994	189		53	23		9	33	27
Ledingham	1988	190	<90	51	14		39	163	24
Lingnau_C	1997	191	T	38	15		10	82	12
Lingnau_T	1997	191	T	36	16		9	80	11
McClelland	1990	192		47	23	L	9	15	60
Oudhuis	2011	207		62	21		32	124	26
Palomar_1	1997	193	Lib	47	18		14	50	29
Pneumatikos	2002	194	T	39	18		5	31	16
Pugin	1991	195	Lib, T	45	16	L	10	38	26
Rocha	1992	196	Lib, T	43	15		27	74	36
Sanchez-Garcia	1998	197		55	26		51	131	39
Stoutenbeek	1996	198	Lib		11		2	49	4
Stoutenbeek '07	2007	199	T	38	15		42	201	21
van der Geest	2016	208	<90	60	22	L	100	301	33
van der Geest	2014	208		58	21	L	29	113	26
Van Meenen	2018	209		66	24		135	455	30
Van Meenen	2018	209		65	23		137	467	29
Veelo	2008	210		61	19		33	231	14
Verwaest PTA	1997	200		56	18		31	200	16
Verwaest OA	1997	200	Lib	57	18		47	220	21
Wiener	1995	201		60	26		11	30	37
Winter	1992	201		58	15	L	33	91	36
Wittekamp PTNy	2018	203		63	22 [e]		645	2082	31
Wittekamp PTNy	2018	203		62	21 [e]		685	2224	31
Wittekamp Chlx	2018	203		61	20 [e]		664	2108	31
Wittekamp PTNy	2018	203		63	22 [e]	L	689	2022	34
Wittekamp PTNy	2018	203		62	21 [e]	L	703	2171	32
Wittekamp Chlx	2018	203		61	20 [e]	L	675	2049	33
Wood	2002	204	T	38	22		3	20	15

Table S3 footnotes

a. Studies [n=50] derived from the following systematic reviews [223-226].

b. Notes; NC – non-concurrent control group; T – Data originating from a study for which the majority of ICU admission were for trauma; <90 – less than 90% of patients received > 24 hours of MV; PP- Control group receiving protocolized parenteral antibiotic prophylaxis; Lib – means data from Liberati [225] see Table s4.
c. Age – Group mean (or median) age (years)
d. Census - L – Late mortality being day 28 or hospital mortality
e. Wittekamp – APACHE II data reported for only 5 of 13 sites

Data for MacNaughton [S165] obtained as abstracted as a personal communication within Chan EY, Ruest A, Meade MO, Cook DJ. Oral decontamination for prevention of pneumonia in mechanically ventilated adults: systematic review and meta-analysis. *BMJ*. 2007;334(7599):889.

Data for Koeman [S161], which is a three-arm study used in the analysis here from [Melsen WG, Rovers MM, Groenwold RH, Bergmans DC, Camus C, Bauer TT, Hanisch EW, Klarin B, Koeman M, Krueger WA, Lacherade JC. Attributable mortality of ventilator-associated pneumonia: a meta-analysis of individual patient data from randomised prevention studies. Lancet Infect Dis 2013;13(8):665-71.] where the two intervention arms [topical Chlorhexidine with or without colistin] are combined as one.

Topical anti-septic intervention regimens were chlorhexidene in various concentrations and preperations in all studies except; povidone-iodine (Chua et al [S156]) and iseganan (Kollef et al [S162]).

SDD/SOD intervention regimens. For the purpose of this analysis, a SDD/SOD regimen was any regimen of topical antibiotic porphylaxis with (SDD) or without (SOD) additional protocolized parenteral antibiotic prophylaxis. SOD regiemns included the following;

- PGV (P, topical polymyxin; G, topical gentamicin; V, topical vancomycin),
- PNeoNal (P, topical polymyxin; Neo, topical neomyxin; Nal = Nalidixic acid),
- PT (P, topical polymyxin; T, topical tobramycin),
- PTChM (P, topical polymyxin; T, topical tobramycin; Ch, topical chlorhexidene; topical mupirocin),
- NorNys (Nor, Norfloxacin; Nys, Nystatin),
- Cz(Ae) = Aerosolized ceftazidime;
- PTA (P, topical polymyxin; T, topical tobramycin; A, topical amphotericin).
- PGA (P, topical polymyxin; G, topical gentamicin; A, topical amphotericin),
- P (P = polymyxin either aerosolized or topical),
- G = gentamicin,
- ChC (Ch, topical chlorhexidine; C, topical colistin),
- PTAM (P, topical polymyxin; T, topical tobramycin; A, topical amphotericin; topical mupirocin),
- PNeoVan (P, topical polymyxin; Neo, topical neomycin; Van, topical vancomycin),
- PGA (P, topical polymyxin; G, topical gentamicin; A, topical amphotericin),
- PGNy (P, topical polymyxin; G, topical gentamicin; Ny, topical nystatin).

SDD regiemns included the following;

- PTA-Ctx (P, topical polymyxin; T, topical tobramycin; A, topical amphotericin; Ctx, parenteral cephalosporin),
- PNoA-Ctx (P, topical polymyxin; No, topical norfloxacin; A, topical amphotericin; Ctx, parenteral cephalosporin),
- PGNy-Ctx (P, topical polymyxin; G, topical gentamicin; Ny, topical nystatin; Ctx, parenteral cephalsporin),
- PTNy-Ctx (P, topical polymyxin; T, topical tobramycin; Ny, topical nystatin; Ctx, parenteral cephalsporin),
- PNA-AmClav (P, topical polymyxin; N, topical netilmicin; A, topical amphotericin; Ctx, parenteral amoxicillin-clavulinate),

- PG-Cip (P, topical polymyxin; G, topical gentamicin; Cip, parenteral ciprofloxacin),
- GA- AmClav (G, topical gentamicin; A, topical amphotericin),
- PCA-Cip (P, topical polymyxin; C, topical ciprofloxacin; A, topical amphotericin; Cip, parenteral ciprofloxacin),
- PTA-Cip (P, topical polymyxin; T, topical tobramycin; A, topical amphotericin; Cip, parenteral ciprofloxacin),
- PTA-Cef (P, topical polymyxin; T, topical tobramycin; A, topical amphotericin; Ctx, parenteral cefuroxime),
- PGA-Ctx (P, topical polymyxin; G, topical gentamicin; A, topical amphotericin; Ctx, parenteral ceftriaxone),
- PTAV-Ctx (P, topical polymyxin; T, topical tobramycin; A, topical amphotericin; Van, topical vancomycin; Ctx, parenteral cephalosporin),
- PNoA-Tri (P, topical polymyxin; No, topical norfloxacin; A, topical amphotericin; trimethoprim),
- OA-O (O = topical ofloxacin, A = topical amphotericin; O = parenteral ofloxacin),
- PTA-Cz (P, topical polymyxin; T, topical tobramycin; A, topical amphotericin; Ctx, parenteral cephalosporin).

Table S4: On treatment [OT] versus intention to treat [ITT] mortality data discrepancies [a]

author	Year	Ref	Intention to treat [ITT] [S225]			On treatment [OT] [S226]		
			Mortality (n)	Patients (n)	Mortality (%)	Mortality (n)	Patients (n)	Mortality (%)
control groups								
Aerdts	1991	173	12	60	20	4	39	10.3
Boland	1991	176	4	32	12.5	4	15	26.7
Hammond	1992	183	31	160	19.3	21	126	16.7
Palomar	1997	193	14	49	28.6	13	42	31
Pugin	1991	195	11	41	26.8	7	27	25.9
Rocha	1992	196	40	77	51.9	24	50	48
Stoutenbeek	1996	198	8	42	19	8	29	27.6
Verwaest	1997	200	40	220	18.2	31	185	16.8
Intervention groups								
Aerdts	1991	173	4	28	14.3	4	18	22.2
Boland	1991	176	2	32	6.3	2	15	13.3
Hammond	1992	183	34	162	21	21	114	18.4
Palomar	1997	193	14	50	28	10	41	24.4
Pugin	1991	195	10	38	26.3	7	25	28.0
Rocha	1992	196	27	74	36.5	10	47	21.3
Stoutenbeek	1996	198	2	49	4	2	30	6.7
Verwaest OA-O	1997	200	47	220	21.4	34	193	17.6
**Effect size**			**Odds ratio**			**Risk ratio**		
Aerdts	1991	173	0.67	0.19 - 2.29		2.17	0.61 - 7.7	
Boland	1991	176	0.47	0.08 - 2.75		0.5	0.11 - 2.33	
Hammond	1992	183	1.11	0.64 - 1.91		1.11	0.64 - 1.91	
Palomar	1997	193	0.97	0.41 - 2.33		0.79	0.39 - 1.59	
Pugin	1991	195	0.97	0.36 - 2.65		1.08	0.44 - 2.64	
Rocha	1992	196	0.53	0.28 - 1.02		0.48	0.26 - 0.89	
Stoutenbeek	1996	198	0.94	0.58 - 1.51		0.24	0.06 - 1.04	
Verwaest OA-O	1997	200	1.22	0.76 - 1.96		1.05	0.68 - 1.64	

a. The data abstracted in Liberati [S225] is intention to treat data [ITT] obtained after personal communications for 25 of 36 studies from the original study authors versus Minozzi [S226] who

abstracted on treatment [OT] data from all 41 published articles. Note that the numerator and denominator counts and the percentages are generally higher for the ITT data [listed in Table S4] due to the common practice of excluding patients from control and intervention arms that had died before completing the four TAP considered necessary to achieve digestive decontamination.

References

S1. Akca O, Koltka K, Uzel S, et al: Risk factors for early-onset, ventilator-associated pneumonia in critical care patients: Selected multiresistant versus nonresistant bacteria. *Anesthesiol* 2000; 93:638–645

S2. Apostolopoulou E, Bakakos P, Katostaras T, et al: Incidence and risk factors for ventilator-associated pneumonia in 4 multidisciplinary intensive care units in Athens, Greece. *Respir Care* 2003; 48:681–688

S3. Artinian V, Krayem H, DiGiovine B. Effects of early enteral feeding on the outcome of critically ill mechanically ventilated medical patients. *Chest*. 2006;129(4):960-7.

S4. Bonten MJ, Bergmans DC, Ambergen AW, et al: Risk factors for pneumonia, and colonization of respiratory tract and stomach in mechanically ventilated ICU patients. *Am J Respir Crit Care Med* 1996; 154:1339–1346

S5. Boots RJ, Lipman J, Bellomo R, Stephens D, Heller RF. Disease risk and mortality prediction in intensive care patients with pneumonia. Australian and New Zealand practice in intensive care (ANZPIC II). *Anesthes and Intensive Care*. 2005;33(1):101.

S6. Cavalcanti M, Ferrer M, Ferrer R, Morforte R, Garnacho A, Torres A: Risk and prognostic factors of ventilator-associated pneumonia in trauma patients. *Crit Care Med.* 2006;34:1067-1072

S7. Cendrero JA, Solé-Violán J, Benitez AB, Catalán JN, Fernández JA, Santana PS, de Castro FR: Role of different routes of tracheal colonization in the development of pneumonia in patients receiving mechanical ventilation. *Chest*. 1999;116:462-470

S8. Chevret S, Hemmer M, Carlet J: Incidence and risk factors of pneumonia acquired in intensive care units. Results from a multicenter prospective study on 996 patients. European Cooperative Group on Nosocomial Pneumonia. Intensive Care Med. 1993;19:256-264

S9. Cook DJ, Walter SD, Cook RJ, Griffith LE, Guyatt GH, Leasa D, Jaeschke RZ, Brun-Buisson C. Incidence of and risk factors for ventilator-associated pneumonia in critically ill patients. *Ann intern med.* 1998;129(6):433-40.

S10. Craven DE, Kunches LM, Kilinsky V, et al. Risk factors for pneumonia and fatality in patients receiving continuous mechanical ventilation. *Am Rev Respir Dis* 1986;133:792-6.

S11. Eddleston JM, Pearson RC, Holland J, Tooth JA, Vohra A, Doran BH. Prospective endoscopic study of stress erosions and ulcers in critically ill adult patients treated with either sucralfate or placebo. *Crit Care Med* 1994;22:1949-54

S12. Ensminger SA, Wright RS, Baddour LM, Afess B: Suspected ventilator-associated pneumonia in cardiac patients admitted to the coronary care unit. Mayo Clin Proc. 2006;81:32–35

S13. Ertugrul BM, Yildirim A, Ay P, Oncu S, Cagatay A, Cakar N, Ertekin C, Ozsut H, Eraksoy H, Calangu S. Ventilator-associated pneumonia in surgical emergency intensive care unit. Saudi med J. 2006;27(1):52.

S14. Fagon JY, Chastre J, Vuagnat A, et al: Nosocomial pneumonia and mortality among patients in intensive care units. *JAMA* 1996; 275:866–869

S15. Fieselmann JF, Bock MJ, Hendryx MS, Wakefield D, Helms CM, Bentler SE: Mechanical ventilation in rural ICUs. *Crit Care* 1999, 3:23-31.

S16. François B, Jafri HS, Chastre J, Sánchez-García M, Eggimann P, Dequin PF, Huberlant V, Soria LV, Boulain T, Bretonnière C, Pugin J. Efficacy and safety of suvratoxumab for prevention of Staphylococcus aureus ventilator-associated pneumonia (SAATELLITE): a multicentre, randomised, double-blind, placebo-controlled, parallel-group, phase 2 pilot trial. *Lancet Infect Dis*. 2021;21(9):1313-23.

S17. Hart R, McNeill S, Maclean S, Hornsby J, Ramsay S. The prevalence of suspected ventilator-associated pneumonia in Scottish intensive care units. *J Intens Care Soc.* 2020;21(2):140-7.

S18. Hébert PC, Wells G, Blajchman MA, Marshall J, Martin C, Pagliarello G, Yetisir E: A multicenter, randomized, controlled clinical trial of transfusion requirements in critical care. *New Engl J Med* 1999, 340:409-417.

S19. Heyland DK, Cook DJ, Schoenfeld PS, Frietag A, Varon J, Wood G: The effect of acidified enteral feeds on gastric colonization in critically ill patients: results of a multicenter randomized trial. Canadian Critical Care Trials Group. *Crit Care Med*. 1999;27:2399-2406

S20. Hyllienmark P, Brattström O, Larsson E, Martling CR, Petersson J, Oldner A: High incidence of post-injury pneumonia in intensive care-treated trauma patients. *Acta Anaesthesiologica Scandinavica.* 2013;57(7):848-54.

S21. Ibrahim EH, Ward S, Sherman G, et al: A comparative analysis of patients with early-onset vs late-onset nosocomial pneumonia in the ICU setting. *Chest* 2000; 117:1434–1442
S22. Ibrahim EH, Tracy L, Hill C, et al: The occurrence of ventilator-associated pneumonia in a community hospital: Risk factors and clinical outcomes. *Chest* 2001; 120:555–561
S23. Ibrahim EH, Mehringer L, Prentice D, et al: Early versus late enteral feeding of mechanically ventilated patients: Results of a clinical trial. *JPEN J Parenter Enteral Nutr* 2002; 26:174–181
S24. Jacobs S, Chang RW, Lee B, Bartlett FW: Continuous enteral feeding: a major cause of pneumonia among ventilated intensive care unit patients. *JPEN J Parenter Enteral Nutr* 1990, 14:353-6.
S25. Jaimes F, De La Rosa G, Gómez E, Múnera P, Ramírez J, Castrillón S. Incidence and risk factors for ventilator-associated pneumonia in a developing country Where is the difference? *Respir Med* 2007;101:762–767.
S26. Kasuya Y, Hargett JL, Lenhardt R, Heine MF, Doufas AG, Remmel KS, Ramirez JA, Akça O. Ventilator-associated pneumonia in critically ill stroke patients: frequency, risk factors, and outcomes. *J crit care*. 2011;26(3):273-9.
S27. Kirschenbaum L, Azzi E, Sfeir T, et al. Effect of continuous lateral rotational therapy on the prevalence of ventilator-associated pneumonia in patients requiring long-term ventilatory care. *Crit Care Med* 2002;30:1983-6.
S28. Ko HK, Yu WK, Lien TC, Wang JH, Slutsky AS, Zhang H, Kou YR. Intensive care unit-acquired bacteremia in mechanically ventilated patients: clinical features and outcomes. *PloS one*. 2013;8(12):e83298.
S29. Kollef MH: Ventilator-associated pneumonia. A multivariate analysis. *JAMA* 1993; 270:1965–1970
S30. Kollef MH, Silver P, Murphy DM, et al: The effect of late-onset ventilator-associated pneumonia in determining patient mortality. *Chest* 1995; 108:1655–1662
S31. Kollef MH, Shapiro SD, Von Harz B, Prentice D, John RS, Silver P, Trovillion E. Patient transport from intensive care increases the risk of developing ventilator-associated pneumonia. *Chest*. 1997;112(3):765-73.
S32. Laupland KB, Kirkpatrick AW, Church DL, Ross T, Gregson DB Intensive-care-unit-acquired bloodstream infections in a regional critically ill population. *J Hosp Infect* 2004;58(2): 137-145.
S33. León C, Ruiz-Santana S, Saavedra P, Almirante B, Nolla-Salas J, Álvarez-Lerma F, José Garnacho-Montero, EPCAN Study Group. (2006) A bedside scoring system ("*Candida* score") for early antifungal treatment in non-neutropenic critically ill patients with *Candida* colonization. *Crit Care Med* 34(3):730-737.
S34. León C, Ruiz-Santana S, Saavedra P, Galván B, Blanco A, Castro C, Carina Balasini Cava Study Group. Usefulness of the "*Candida* score" for discriminating between *Candida* colonization and invasive candidiasis in non-neutropenic critically ill patients: a prospective multicenter study. *Crit Care Med* 2009; 37:1624-1633.
S35. Luna CM, Blanzaco D, Niederman MS, Matarucco W, Baredes NC, Desmery P, Palizas F, Menga G, Rios F, Apezteguia C. Resolution of ventilator-associated pneumonia: prospective evaluation of the clinical pulmonary infection score as an early clinical predictor of outcome. *Crit Care Med.* 2003;31(3):676-82.
S36. Luna CM, Aruj P, Niederman MS, Garzon J, Violi D, Prignoni A, Rios F, Baquero S, Gando S. Appropriateness and delay to initiate therapy in ventilator-associated pneumonia. *Eur Respir J*. 2006;27:158-64.
S37. Magnason S, Kristinsson KG, Stefansson T, Erlendsdottir H, Jonsdottir K, Kristjansson M, Gudmundsson S: Risk factors and outcome in ICU-acquired infections. *Acta Anaesthesiol Scandinavica.* 2008;52:1238-1245
S38. Muscedere J, Sinuff T, Heyland DK, Dodek PM, Keenan SP, Wood G, Jiang X, Day AG, Laporta D, Klompas M, Canadian Critical Care Trials Group. The clinical impact and preventability of ventilator-associated conditions in critically ill patients who are mechanically ventilated. *Chest*. 2013;144(5):1453-60.
S39. Nguyen NQ, Fraser RJ, Bryant LK, Burgstad C, Chapman MJ, Bellon M, Wishart J, Holloway RH, Horowitz M. The impact of delaying enteral feeding on gastric emptying, plasma cholecystokinin, and peptide YY concentrations in critically ill patients. *Crit Care Med*. 2008;36(5):1469-74.
S40. Osmon S, Warren D, Seiler SM, Shannon W, Fraser VJ, Kollef MH: The influence of infection on hospital mortality for patients requiring >48 h of intensive care. *Chest* 2003, 124:1021-1029.
S41. Petri MG, König J, Moecke HP, Gramm HJ, Barkow H, Kujath P, Dennhart R, Lode H (1997) Epidemiology of invasive mycosis in ICU patients: a prospective multicenter study in 435 non-neutropenic patients. *Intensive Care Med* 23(3):317-325.
S42. Ramirez P, Lopez-Ferraz C, Gordon M, Gimeno A, Villarreal E, Ruiz J, Menendez R, Torres A. From starting mechanical ventilation to ventilator-associated pneumonia, choosing the right moment to start antibiotic treatment. *Critical Care*. 2016;20(1):1-7.
S43. Rello J, Lorente C, Diaz E, et al. Incidence, etiology, and outcome of nosocomial pneumonia in ICU patients requiring percutaneous tracheotomy for mechanical ventilation. *Chest*. 2003;124:2239-2243.
S44. Rodrigues PM, Carmo Neto ED, Santos LR, Knibel MF. Ventilator-associated pneumonia: epidemiology and impact on the clinical evolution of ICU patients. *J brasileiro de pneumologia*. 2009;35(11):1084-91.
S45. Ruiz M, Torres A, Ewig S, Marcos MA, Alcón A, Lledó R, Asenjo MA, Maldonaldo A. Noninvasive versus invasive microbial investigation in ventilator-associated pneumonia: evaluation of outcome. *Am J Respir Crit Care Med*. 2000;162(1):119-25.
S46. Schweickert WD, Gehlbach BK, Pohlman AS, Hall JB, Kress JP. Daily interruption of sedative infusions and complications of critical illness in mechanically ventilated patients. *Crit Care Med*. 2004;32(6):1272-6.

S47. Shahin J, Bielinski M, Guichon C, Flemming C, Kristof AS Suspected ventilator-associated respiratory infection in severely ill patients: a prospective observational study. *Crit Care* 2013;17(5): R251
S48. Sinuff T, Muscedere J, Cook D, Dodek P, Heyland D, Canadian Critical Care Trials Group. Ventilator-associated pneumonia: Improving outcomes through guideline implementation. *J Crit care*. 2008;23(1):118-25.
S49. Sofianou DC, Constandinidis TC, Yannacou M, Anastasiou H, Sofianos E: Analysis of risk factors for ventilator-associated pneumonia in a multidisciplinary intensive care unit. *Eur J Clin Microbiol Infect Dis* 2000, 19:460-463.
S50. Steen J, Vansteelandt S, De Bus L, Depuydt P, Gadeyne B, Benoit DD, Decruyenaere J. Attributable mortality of ventilator-associated pneumonia. Replicating findings, revisiting methods. *Annals Am Thor Soc.* 202;18(5):830-7.
S51. Suka M, Yoshida K, Uno H, Takezawa J. Incidence and outcomes of ventilator-associated pneumonia in Japanese intensive care units: the Japanese nosocomial infection surveillance system. *Infection Control & Hosp Epidemiol.* 2007;28(3):307-13.
S52. Tan X, Zhu S, Yan D, Chen W, Chen R, Zou J, Yan J, Zhang X, Farmakiotis D, Mylonakis E. Candida spp. airway colonization: A potential risk factor for Acinetobacter baumannii ventilator-associated pneumonia. *Sabouraudia*. 2016;54(6):557-66.
S53. Tejada Artigas AT, Dronda SB, Vallés EC, Marco JM, Usón MC, Figueras P, Suarez FJ, Hernandez A: Risk factors for nosocomial pneumonia in critically ill trauma patients. *Crit Care Med*. 2001;29:304-9.
S54. Timsit JF, Chevret S, Valcke J, et al. Mortality of nosocomial pneumonia in ventilated patients: influence of diagnostic tools. *Am J Respir Crit Care Med* 1996; 154: 116-23.
S55. Urli T, Perone G, Acquarolo A, Zappa S, Antonini B, Ciani A: Surveillance of infections acquired in intensive care: usefulness in clinical practice. *J Hosp Infect* 2002, 52:130-5.
S56. van der Kooi TI, de Boer AS, Manniën J, Wille JC, Beaumont MT, Mooi BW, van den Hof S. Incidence and risk factors of device-associated infections and associated mortality at the intensive care in the Dutch surveillance system. *Intensive Care Med* 2007;33(2):271.
S57. Violan JS, Sanchez-Ramirez C, Mujica AP, Cendrero JC, Fernandez JA, de Castro FR. Impact of nosocomial pneumonia on the outcome of mechanically-ventilated patients. *Crit Care (Lond)* 1998;2:19-23.
S58. Violan JS, Fernandez JA, Benítez AB, Cendrero JA, De Castro FR. Impact of quantitative invasive diagnostic techniques in the management and outcome of mechanically ventilated patients with suspected pneumonia. *Crit Care Med* 2000;28(8):2737-41.
S59. Walsh TS, Boyd JA, Watson D, Hope D, Lewis S, Krishan A, Forbes JF, Ramsay P, Pearse R, Wallis C, Cairns C. Restrictive versus liberal transfusion strategies for older mechanically ventilated critically ill patients: a randomized pilot trial. *Crit Care Med* 2013;41(10):2354-63.
S60. Warren DK, Shukla SJ, Olsen MA, et al. Outcome and attributable cost of ventilator-associated pneumonia among intensive care unit patients in a suburban medical center. *Crit Care Med* 2003;31:1312-7.
S61. Woske HJ, Röding T, Schulz I, Lode H. Ventilator-associated pneumonia in a surgical intensive care unit Epidemiology, etiology and comparison of three bronchoscopic methods for microbiological specimen sampling. *Crit Care* 2001;5:167–173.
S62. Xie J, Li S, Xue M, Yang C, Huang Y, Chihade DB, Liu L, Yang Y, Qiu H. Early-and Late-Onset Bloodstream Infections in the Intensive Care Unit: A Retrospective 5-Year Study of Patients at a University Hospital in China. *J Infect Dis*. 2020;221(Supplement_2):S184-92.
S63. Zygun DA, Zuege DJ, Boiteau PJ, Lapland KB, Henderson EA, Kortbeek JB, Doig CJ. Ventilator-associated pneumonia in severe traumatic brain injury. *Neurocritical care*. 2006;5(2), 108-114.
S64. Bloos F, Müller S, Harz A, et al.Effects of staff training on the care of mechanically ventilated patients: A prospective cohort study. *Br J Anaesth* 2009; 103:232237
S65. Crunden E, Boyce C, Woodman H, et al.An evaluation of the impact of the ventilator care bundle. *Nurs Crit Care* 2005; 10:242246
S66. DeLuca LA Jr, Walsh P, Davidson DD Jr, et al.Impact and feasibility of an emergency department-based ventilator-associated pneumonia bundle for patients intubated in an academic emergency department. *Am J Infect Control* 2017; 45:151157
S67. Hawe CS, Ellis KS, Cairns CJ, et al.Reduction of ventilator-associated pneumonia: Active versus passive guideline implementation. *Intensive Care Med* 2009; 35:11801186
S68. Morris AC, Hay AW, Swann DG, et al.Reducing ventilator-associated pneumonia in intensive care: Impact of implementing a care bundle. *Crit Care Med* 2011; 39:22182224
S69. Parisi M, Gerovasili V, Dimopoulos S, et al.Use of ventilator bundle and staff education to decrease ventilator-associated pneumonia in intensive care patients. *Crit Care Nurse* 2016; 36:e1e7
S70. Acosta-Escribano J, Fernández-Vivas M, Carmona TG, Caturla-Such J, Garcia-Martinez M, Menendez-Mainer A, Sanchez-Payá J (2010) Gastric versus transpyloric feeding in severe traumatic brain injury: a prospective, randomized trial. *Intensive Care Med* 36:1532-1539

S71. Altintas ND, Aydin K, Türkoğlu MA, Abbasoğlu O, Topeli A. Effect of enteral versus parenteral nutrition on outcome of medical patients requiring mechanical ventilation. *Nutrition in Clinical Practice* 2011;26(3):322-9.

S72. Arabi YM, Aldawood AS, Haddad SH, Al-Dorzi HM, Tamim HM, Jones G, Mehta S, McIntyre L, Solaiman O, Sakkijha MH, Sadat M. Permissive underfeeding or standard enteral feeding in critically ill adults. *N Engl J Med*. 2015;372(25):2398-408.

S73. Ben-Menachem T, Fogel R, Patel RV, et al. Prophylaxis for stress-related gastric hemorrhage in the medical intensive care unit. A randomized, controlled, single-blind study. *Ann Intern Med* 1994;121:568-75.

S74. Boivin MA, Levy H. Gastric feeding with erythromycin is equivalent to transpyloric feeding in the critically ill. *Crit Care Med* 2001;29(10):1916-9.

S75. Bonten MJ, Gaillard CA, Van der Geest S, Van Tiel FH, Beysens AJ, Smeets HG, Stobberingh EE: The role of intragastric acidity and stress ulcer prophylaxis on colonization and infection in mechanically ventilated ICU patients. A stratified, randomized, double-blind study of sucralfate versus antacids. *Am J Respir Crit Care Med.* 1995;152:1825-1834.

S76. Bonten MJ, Gaillard CA, Van der Hulst R, De Leeuw PW, Van Der Geest S, Stobberingh EE, Soeters PB. Intermittent enteral feeding: the influence on respiratory and digestive tract colonization in mechanically ventilated intensive-care-unit patients. *Am J Respir Crit Care Med*. 1996;154(2):394-9.

S77. Conrad SA, Gabrielli A, Margolis B, Quartin A, Hata JS, Frank WO, et al. Randomized, double-blind comparison of immediate-release omeprazole oral suspension versus intravenous cimetidine for the prevention of upper gastrointestinal bleeding in critically ill patients. *Crit Care Med* 2005;33(4):760-5.

S78. Cook D, Guyatt G, Marshall J, et al A comparison of sucralfate and ranitidine for the prevention of upper gastrointestinal bleeding in patients requiring mechanical ventilation. Canadian Critical Care Trials Group. *N Engl J Med* 1998;338:791-797

S79. Davies AR, Froomes PR, French CJ, Bellomo R, Gutteridge GA, Nyulasi I, etal. Randomized comparison of nasojejunal and nasogastric feeding in critically ill patients. *Crit Care Med* 2002;30(3):586-90.

S80. Davies AR, Morrison SS, Bailey MJ, Bellomo R, Cooper DJ, Doig GS, Finfer SR, Heyland DK: A multicenter, randomized controlled trial comparing early nasojejunal with nasogastric nutrition in critical illness. *Crit Care Med* 2012;40:2342-2348

S81. Driks MR, Craven DE, Celli BR, et al (1987) Nosocomial pneumonia in intubated patients given sucralfate as compared with antacids or histamine type 2 blockers. The role of gastric colonization. *N Engl J Med* 317:1376-1382

S82. Eddleston JM, Vohra A, Scott P, et al. A comparison of the frequency of stress ulceration and secondary pneumonia in sucralfate- or ranitidine-treated intensive care unit patients. *Crit Care Med* 1991;19:1491-6.

S83. Esparza J, Boivin MA, Hartshorne MF, Levy H. Equal aspiration rates in gastrically and transpylorically fed critically ill patients. *Intens Care Med* 2001;27(4):660-4.

S84. Fabian TC, Boucher BA, Croce MA, Kuhl DA, Janning SW, Coffey BC, Kudsk KA: Pneumonia and stress ulceration in severely injured patients: a prospective evaluation of the effects of stress ulcer prophylaxis. *Arch Surg*. 1993;128(2):185-92.

S85. Grau T, Bonet A, Miñambres E, Piñeiro L, Irles JA, Robles A, Acosta J, Herrero I, Palacios V, Lopez J, Blesa A. The effect of L-alanyl-L-glutamine dipeptide supplemented total parenteral nutrition on infectious morbidity and insulin sensitivity in critically ill patients. *Crit Care Med*. 2011;39(6):1263-8.

S86. Grau-Carmona T, Morán-García V, García-de-Lorenzo A, Heras-de-la-Calle G, Quesada-Bellver B, López-Martínez J, González-Fernández C, Montejo-González JC, Blesa-Malpica A, Albert-Bonamusa I, Bonet-Saris A. Effect of an enteral diet enriched with eicosapentaenoic acid, gamma-linolenic acid and anti-oxidants on the outcome of mechanically ventilated, critically ill, septic patients. *Clin nutrition*. 2011;30(5):578-84.

S87. Hanisch EW, Encke A, Naujoks F, et al. A randomized, double-blind trial for stress ulcer prophylaxis shows no evidence of increased pneumonia. *Am J Surg* 1998;176:453-7.

S88. Harvey SE, Parrott F, Harrison DA, Bear DE, Segaran E, Beale R, Bellingan G, Leonard R, Mythen MG, Rowan KM. Trial of the route of early nutritional support in critically ill adults. *N Engl J Med*. 2014;371(18):1673-84.

S89. Heidegger CP, Berger MM, Graf S, Zingg W, Darmon P, Costanza MC, Thibault R, Pichard C. Optimisation of energy provision with supplemental parenteral nutrition in critically ill patients: a randomised controlled clinical trial. *The Lancet.* 2013;381(9864):385-93.

S90. Heyland DK, Cook DJ, Schoenfeld PS, Frietag A, Varon J, Wood G The effect of acidified enteral feeds on gastric colonization in critically ill patients: results of a multicenter randomized trial. Canadian Critical Care Trials Group. *Crit Care Med* 1999;27:2399-2406

S91. Heyland DK, Murch L, Cahill N, McCall M, Muscedere J, Stelfox HT, Bray T, Tanguay T, Jiang X, Day AG. Enhanced protein-energy provision via the enteral route feeding protocol in critically ill patients: results of a cluster randomized trial. *Crit Care Med*. 2013;41(12):2743-53.

S92. Hsu CW, Sun SF, Lin SL, Kang SP, Chu KA, Lin CH, et al. Duodenal versus gastric feeding in medical intensive care unit patients. *Crit Care Med* 2009;37(6):1866-72.

S93. Ibrahim EH, Mehringer L, Prentice D, et al: Early versus late enteral feeding of mechanically ventilated patients: Results of a clinical trial. *JPEN J Parenter Enteral Nutr* 2002; 26:174–181
S94. Johnstone J, Meade M, Lauzier F, Marshall J, Duan E, Dionne J, Arabi YM, Heels-Ansdell D, Thabane L, Lamarche D, Surette M. Effect of probiotics on incident ventilator-associated pneumonia in critically Ill patients: a randomized clinical trial. *JAMA*. 2021;326(11):1024-33.
S95. Kantorova I, Svoboda P, Scheer P, Doubek J, Rehorkova D, Bosakova H, et al. Stress ulcer prophylaxis in critically ill patients: a randomized controlled trial. *Hepato-gastroenterol* 2004;51(57):757-61.
S96. Kearns PJ, Chin D, Mueller L, Wallace K, Jensen WA, Kirsch CM. The incidence of ventilator-associated pneumonia and success in nutrient delivery with gastric versus small intestinal feeding: a randomized clinical trial. *Crit Care Med* 2000;28:1742-1746
S97. Klarin B, Molin G, Jeppsson B, Larsson A. Use of the probiotic Lactobacillus plantarum 299 to reduce pathogenic bacteria in the oropharynx of intubated patients: a randomised controlled open pilot study. *Crit Care* 2008;12(6):R136.
S98. Knight DJ, Gardiner D, Banks A, Snape SE, Weston VC, Bengmark S, Girling KJ: Effect of synbiotic therapy on the incidence of ventilator associated pneumonia in critically ill patients: a randomised, double-blind, placebo-controlled trial. *Intensive Care Med.* 2009;35:854-861.
S99. Kortbeek JB, Haigh PI, Doig C. Duodenal versus gastric feeding in ventilated blunt trauma patients: a randomized controlled trial. *J Trauma* 1999;46:992-6.
S100. Kotzampassi K, Giamarellos-Bourboulis EJ, Voudouris A, Kazamias P, Eleftheriadis E: Benefits of a synbiotic formula (Synbiotic 2000Forte) in critically ill trauma patients: early results of a randomized controlled trial. *World J Surg* 2006;30:1848-1855.
S101. Levy MJ, Seelig CB, Robinson NJ, Ranney JE. Comparison of omeprazole and ranitidine for stress ulcer prophylaxis. *Digestive Diseases and Sciences* 1997;42(6):1255-9.
S102. Martin LF, Booth FV, Karlstadt RG, et al. Continuous intravenous cimetidine decreases stress-related upper gastrointestinal hemorrhage without promoting pneumonia. *Crit Care Med* 1993;21:19–30
S103. Montecalvo MA, Steger KA, Farber HW: Nutritional outcome and pneumonia in critical care patients randomized to gastric versus jejunal tube feedings. The Critical Care Research Team. *Crit Care Med* 1992, 20:1377-1387.
S104. Montejo JC, Grau T, Acosta J, Ruiz-Santana S, Planas M, García-de-Lorenzo A, López-Martínez J. Multicenter, prospective, randomized, single-blind study comparing the efficacy and gastrointestinal complications of early jejunal feeding with early gastric feeding in critically ill patients. *Crit Care Med* 2002;30(4):796-800.
S105. Morrow LE, Kollef MH, Casale TB: Probiotic prophylaxis of ventilator-associated pneumonia: a blinded, randomized, controlled trial. *Am J Respir Crit Care Med* 2010; 182:1058-1064
S106. Muscedere J, Maslove DM, Boyd JG, O'Callaghan N, Sibley S, Reynolds S, Albert M, Hall R, Jiang X, Day AG, Jones G. Prevention of nosocomial infections in critically ill patients with lactoferrin: a randomized, double-blind, placebo-controlled study. *Crit Care Med*. 2018 ;46(9):1450-6.
S107. Nguyen NQ, Fraser RJ, Bryant LK, Burgstad C, Chapman MJ, Bellon M, et al. The impact of delaying enteral feeding on gastric emptying, plasma cholecystokinin, and peptide YY concentrations in critically ill patients. *Crit Care Med* 2008;36(5):1469-74.
S108. O'Keefe GE, Gentilello LM, Maier RV. Incidence of infectious complications associated with the use of histamine2-receptor antagonists in critically ill trauma patients. *Ann Surg* 1998;227:120-5.
S109. Pickworth KK, Falcone RE, Hoogeboom JE, et al Occurrence of nosocomial pneumonia in mechanically ventilated trauma patients: a comparison of sucralfate and ranitidine. *Crit Care Med* 1993;21:1856-1862
S110. Prod'hom G, Leuenberger P, Koerfer J, Blum A, Chiolero R, Schaller MD, et al. Nosocomial pneumonia in mechanically ventilated patients receiving antacid, ranitidine, or sucralfate as prophylaxis for stress ulcer. A randomized controlled trial. *Ann Intern Med* 1994;120(8):653-62.
S111. Rice TW, Mogan S, Hays MA, Bernard GR, Jensen GL, Wheeler AP. A randomized trial of initial trophic versus full-energy enteral nutrition in mechanically ventilated patients with acute respiratory failure. *Crit Care Med* 2011;39(5):967.
S112. Ruiz-Santana S, Ortiz E, Gonzalez B, Bolanos J, Ruiz-Santana AJ, Manzano JL. Stress-induced gastroduodenal lesions and total parenteral nutrition in critically ill patients: frequency, complications, and the value of prophylactic treatment. A prospective, randomized study. *Crit Care Med* 1991;19(7):887-91.
S113. Spindler-Vesel A, Bengmark S, Vovk I, Cerovic O, Kompan L: Synbiotics, prebiotics, glutamine, or peptide in early enteral nutrition: a randomized study in trauma patients. *J Parenter Enteral Nutr* 2007;31:119-126.
S114. Terzi Coelho CB, Dragosavac D, Coelho Neto JS, Montes CG, Guerrazzi F, Andreollo NA. Ranitidine is unable to maintain gastric pH levels above 4 in septic patients. *J Crit Care* 2009; Vol. 24, issue 4:627.e7-13.
S115. Thomason MH, Payseur ES, Hakenewerth AM, et al. Nosocomial pneumonia in ventilated trauma patients during stress ulcer prophylaxis with sucralfate, antacid, and ranitidine. *J Trauma* 1996;41:503-8.

S116. White H, Sosnowski K,Tran K, Reeves A, Jones M. A randomised controlled comparison of early postpyloric versus early gastric feeding to meet nutritional targets in ventilated intensive care patients. *Crit Care Med* 2009;13(6):1-8.
S117. Wischmeyer PE, Hasselmann M, Kummerlen C, Kozar R, Kutsogiannis DJ, Karvellas CJ, et al. A randomized trial of supplemental parenteral nutrition in underweight and overweight critically ill patients: the TOP-UP pilot trial. *Crit Care* 2017;21:142.
S118. Ahrens T, Kollef M, Stewart J, Shannon W. Effect of kinetic therapy on pulmonary complications. *Am J Crit care*. 2004;13(5):376-82.
S119. Barquist ES, Amortegui J, Hallal A, Giannotti G, Whinney R, Alzamel H, MacLeod J. Tracheostomy in ventilator dependent trauma patients: a prospective, randomized intention-to-treat study. *J Trauma and Acute Care Surg*. 2006 ;60(1):91-7.
S120. Boots RJ, Howe S, George N, et al. Clinical utility of hygroscopic heat and moisture exchangers in intensive care patients. *Crit Care Med* 1997;25:1707-12.
S121. Boots RJ, George N, Faoagali JL, et al. Double-heater-wire circuits and heat-and-moisture exchangers and the risk of ventilator-associated pneumonia. *Crit Care Med* 2006;34:687-93.
S122. Darvas JA, Hawkins LG. The closed tracheal suction catheter: 24 hour or 48 hour change? *Aust Crit Care*. 2003;16:86-92.
S123. David D, Samuel P, David T, Keshava SN, Irodi A, Peter JV. An open-labelled randomized controlled trial comparing costs and clinical outcomes of open endotracheal suctioning with closed endotracheal suctioning in mechanically ventilated medical intensive care patients. *J Crit Care*. 2011;26(5):482-8.
S124. Deppe SA, Kelly JW, Thoi LL, et al. Incidence of colonization, nosocomial pneumonia, and mortality in critically ill patients using a Trach Care closed-suction system versus an open-suction system: prospective, randomized study. *Crit Care Med* 1990;18:1389-93.
S125. Drakulovic MB, Torres A, Bauer TT, Nicolas JM, Nogué S, Ferrer M: Supine body position as a risk factor for nosocomial pneumonia in mechanically ventilated patients: a randomised trial. *Lancet*. 1999;354(9193):1851-1858
S126. Fink MP, Helsmoortel CM, Stein KL, et al. The efficacy of an oscillating bed in the prevention of lower respiratory tract infection in critically ill victims of blunt Trauma A prospective study. *Chest* 1990;97:132-7.
S127. Keeley L. Reducing the risk of ventilator-acquired pneumonia through head of bed elevation. *Nursing in Critical Care* 2007;12(6):287-94.
S128. Kollef MH, Silver P, Murphy DM, Trovillion E: The effect of late-onset ventilator-associated pneumonia in determining patient mortality. *Chest*. 1995;108: 1655-62.
S129. Kollef MH, Vlasnik J, Sharpless L, Pasque C, Murphy D, Fraser V (1997) Scheduled change of antibiotic classes: A strategy to decrease the incidence of ventilator-associated pneumonia. *Am J Respir Crit Care Med* 156:1040–1048
S130. Kollef MH, Shapiro SD, Boyd V, et al. A randomized clinical trial comparing an extended-use hygroscopic condenser humidifier with heated-water humidification in mechanically ventilated patients. *Chest* 1998;113:759-67.
S131. Kollef MH, Afessa B, Anzueto A, Veremakis C, Kerr KM, Margolis BD, Craven DE, Roberts PR, Arroliga AC, Hubmayr RD, Restrepo MI. Silver-coated endotracheal tubes and incidence of ventilator-associated pneumonia: the NASCENT randomized trial. *JAMA*. 2008;300(7):805-13.
S132. Leaf DE, Raed A, Donnino MW, Ginde AA, Waikar SS. Randomized controlled trial of calcitriol in severe sepsis. *Am J Respir Crit Care Med.* 2014;190(5):533-41.
S133. Lorente L, Lecuona M, Málaga J, Revert C, Mora ML, Sierra A: Bacterial filters in respiratory circuits: an unnecessary cost? *Crit Care Med* 2003;31:2126-2130
S134. Lorente L, Lecuona M, Galván R, Ramos MJ, Mora ML, Sierra A: Periodically changing ventilator circuits is not necessary to prevent ventilator-associated pneumonia when a heat and moisture exchanger is used. *Infect Control Hosp Epidemiol.* 2004;25:1077-1082
S135. Lorente L, Lecuona M, Martin MM, et al. Ventilator-associated pneumonia using a closed versus an open tracheal suction system. *Crit Care Med* 2005;33:115-9.
S136. Lorente L, Lecuona M, Jimenez A, Mora ML, Sierra A: Ventilator-associated pneumonia using a heated humidifier or a heat and moisture exchanger: a randomized controlled trial [ISRCTN88724583]. *Crit Care* 2006;10:R116
S137. Lorente L, Lecuona M, Jiménez A, Mora ML, Sierra A: Tracheal suction by closed system without daily change versus open system. *Intensive Care Med.* 2006;32:538-44.
S138. Lorente L, Lecuona M, Jimenez A, Mora ML, Sierra: Influence of an endotracheal tube with polyurethane cuff and subglottic secretion drainage on pneumonia. *Am J Respir Crit Care Med.* 2007;176:1079-1083
S139. Lorente L, Lecuona M, Jiménez A, Lorenzo L, Roca I, Cabrera J, Llanos C, Mora ML: Continuous endotracheal tube cuff pressure control system protects against ventilator-associated pneumonia. *Crit Care.* 2014;18(2):1.

S140. MacIntyre NR, Helms M, Wunderink R, Schmidt G, Sahn SA. Automated rotational therapy for the prevention of respiratory complications during mechanical ventilation. *Respir Care*. 1999;44(12):1447-51.
S141. Mahmoodpoor A, Hamishehkar H, Hamidi M, Shadvar K, Sanaie S, Golzari SE, Khan ZH, Nader ND. A prospective randomized trial of tapered-cuff endotracheal tubes with intermittent subglottic suctioning in preventing ventilator-associated pneumonia in critically ill patients. *J Crit care*. 2017;38:152-6.
S142. Memish ZA, Oni GA, Djazmati W, et al. A randomized clinical trial to compare the effects of a heat and moisture exchanger with a heated humidifying system on the occurrence rate of ventilator-associated pneumonia. *Am J Infect Control* 2001;29:301-5.
S143. Nair P, Venkatesh B, Lee P, Kerr S, Hoechter DJ, Dimeski G, Grice J, Myburgh J, Center JR. A randomized study of a single dose of intramuscular cholecalciferol in critically ill adults. *Crit Care Med*. 2015;43(11):2313-20.
S144. Ntoumenopoulos G, Presneill J, McElholum M, Cade J. Chest physiotherapy for the prevention of ventilator-associated pneumonia. *Intensive Care Med*. 2002;28(7):850-6.
S145. Patman S, Jenkins S, Stiller K. Physiotherapy does not prevent, or hasten recovery from, ventilator-associated pneumonia in patients with acquired brain injury. *Intensive Care Med*. 2009;35(2):258-65.
S146. Rumbak MJ, Truncale T, Newton MN, Adams B, Hazard P. A Prospective, Randomized Study Comparing Early Versus Delayed Percutaneous Tracheostomy In Critically Ill Medical Patients Requiring Prolonged Mechanical Ventilation. *Chest*. 2000;118(4):97S-8S.
S147. Smulders K, van der Hoeven H, Weers-Pothoff I, Vandenbroucke-Grauls C A randomized clinical trial of intermittent subglottic secretion drainage in patients receiving mechanical ventilation. *Chest* 2002;121:858-862
S148. Topeli A, Harmanci A, Cetinkaya Y, et al. Comparison of the effect of closed versus open endotracheal suction systems on the development of ventilator-associated pneumonia. *J Hosp Infect* 2004;58:14-9.
S149. Traver GA, Tyler ML, Hudson LD, Sherrill DL, Quan SF. Continuous oscillation: Outcome in critically III patients. *J Crit care*. 1995;10(3):97-103.
S150. Valencia M, Ferrer M, Farre R, Navajas D, Badia JR, Nicolas JM, Torres A: Automatic control of tracheal tube cuff pressure in ventilated patients in semirecumbent position: a randomized trial. *Crit Care Med*. 2007;35: 1543-9.
S151. Valles J, Artigas A, Rello J, et al. Continuous aspiration of subglottic secretions in preventing ventilator-associated pneumonia. *Ann Intern Med* 1995;122:179-86.
S152. van Nieuwenhoven CA, Vandenbroucke-Grauls C, van Tiel FH, Joore HC, van Schijndel RJ, van der Tweel I, Ramsay G, Bonten MJ. Feasibility and effects of the semirecumbent position to prevent ventilator-associated pneumonia: a randomized study. *Crit care med*. 2006;34(2):396-402.
S153. Young D, Harrison DA, Cuthbertson BH, Rowan K, TracMan Collaborators. Effect of early vs late tracheostomy placement on survival in patients receiving mechanical ventilation: the TracMan randomized trial. *JAMA*. 2013;309(20):2121-9.
S154. Bellissimo-Rodrigues F, Bellissimo-Rodrigues WT, Viana JM, Gil Cezar Alkmim Teixeira MD, Nicolini E, Auxiliadora-Martins M, Martinez R. Effectiveness of oral rinse with chlorhexidine in preventing nosocomial respiratory tract infections among intensive care unit patients. *Infect Control Hosp Epidemiol* 2009;30(10):952-958.
S155. Bellissimo-Rodrigues WT, Menegueti MG, Gaspar GG, Nicolini EA, Auxiliadora-Martins M, Basile-Filho A, Martinez R, Bellissimo-Rodrigues F. Effectiveness of a dental care intervention in the prevention of lower respiratory tract nosocomial infections among intensive care patients: a randomized clinical trial. *Infect Control Hosp Epidemiol*. 2014;35(11):1342-8.
S156. Chua JV, Dominguez EA, Sison CM, Berba RP. The efficacy of povidone-iodine oral rinse in preventing ventilator-associated pneumonia: a randomized, double-blind, placebo-controlled (VAPOR) trial: preliminary report. *Philipp J Microbiol Infect Dis*. 2004;33(153):e161.
S157. Dale CM, Rose L, Carbone S, Pinto R, Smith OM, Burry L, Fan E, Amaral AC, McCredie VA, Scales DC, Cuthbertson BH. Effect of oral chlorhexidine de-adoption and implementation of an oral care bundle on mortality for mechanically ventilated patients in the intensive care unit (CHORAL): a multi-center stepped wedge cluster-randomized controlled trial. *Intensive Care Med*. 2021;47(11):1295-302.
S158. de Lacerda Vidal CF, Vidal AK, Monteiro JG, Cavalcanti A, Henriques AP, Oliveira M, Godoy M, Coutinho M, Sobral PD, Vilela CÂ, Gomes B. Impact of oral hygiene involving toothbrushing versus chlorhexidine in the prevention of ventilator-associated pneumonia: a randomized study. *BMC Infect Dis*. 2017;17(1):1-9.
S159. Fu T, Zhong Q, Zheng C. Bacteriostasis effect of oral administration of chlorhexidine on patients with mechanical ventilation and prevention and treatment of ventilator - associated pneumonia. *Chinese Nursing Research* 2019;33(3):431-4.
S160. Genuit T, Bochicchio G, Napolitano LM, McCarter RJ, Roghman MC. Prophylactic chlorhexidine oral rinse decreases ventilator-associated pneumonia in surgical ICU patients. *Surgical infections*. 2001;2(1):5-18.

S161. Koeman M, van der Ven AJ, Hak E, et al. Oral decontamination with chlorhexidine reduces the incidence of ventilator-associated pneumonia. *Am J Respir Crit Care Med* 2006;173:1348-1355
S162. Kollef M, Pittet D, Sanchez Garcia M, et al. A randomized double-blind trial of iseganan in prevention of ventilator-associated pneumonia. *Am J Respir Crit Care Med* 2006: 173:91-7.
S163. Long Y, Mou G, Zuo Y, lv F, Feng Q, Du J. Effect of modified oral nursing method on the patients with orotracheal intubation. *Journal of Nurses* Training 2012;27(24):2290-3.
S164. Lorente L, Lecuona M, Jimenez A, Palmero S, Pastor E, Lafuente N, et al. Ventilator-associated pneumonia with or without toothbrushing: a randomized controlled trial. *Eur J Clin Microbiol Infect Dis* 2012;31(10):2621-9.
S165. MacNaughton PD, Bailey J, Donlin N, Branfield P, Williams A, Rowswell H. A randomised controlled trial assessing the efficacy of oral chlorhexidine in ventilated patients. European Society of Intensive Care Medicine, 17th Annual Congress, Berlin, Germany. *Intensive Care Med* 2004;30(Suppl),S5-18.
S166. Meinberg MC, Cheade MD, Miranda AL, Fachini MM, Lobo SM. Uso de clorexidina 2% gel e escovação mecânica na higiene bucal de pacientes sob ventilação mecânica: efeitos na pneumonia associada a ventilador. *Revista brasileira de terapia intensiva*. 2010;24(4):369-74.
S167. Özçaka Ö, Başoğlu ÖK, Buduneli N, Taşbakan MS, Bacakoğlu F, Kinane DF. Chlorhexidine decreases the risk of ventilator-associated pneumonia in intensive care unit patients: a randomized clinical trial. *J Periodont Res* 2012;47(5):584-92.
S168. Pobo A, Lisboa T, Rodriguez A, Sole R, Magret M, Trefler S, et al. A randomized trial of dental brushing for preventing ventilator-associated pneumonia. *Chest* 2009;136(2):433-9.
S169. Scannapieco F, Yu J, Raghavendran K, Vacanti A, Owens S, Wood K, Mylotte JA. randomized trial of chlorhexidine gluconate on oral bacterial pathogens in mechanically ventilated patients. *Critical Care*, 2009;13(4): R117.
S170. Swan JT, Ashton CM, Bui LN, Pham VP, Shirkey BA, Blackshear JE, Bersamin JB, Pomer RM, Johnson ML, Magtoto AD, Butler MO. Effect of chlorhexidine bathing every other day on prevention of hospital-acquired infections in the surgical ICU: a single-center, randomized controlled trial. *Crit Care Med.* 2016;44(10):1822-32.
S171. Tantipong H, Morkchareonpong C, Jaiyindee S, Thamlikitkul V. Randomized controlled trial and meta-analysis of oral decontamination with 2% chlorhexidine solution for the prevention of ventilator-associated pneumonia. *Infect Control Hosp Epidemiol* 2008;29:131-136.
S172. Abele-Horn M, Dauber A, Bauernfeind A, Russwurm W, Seyfarth-Metzger I, Gleich P, Ruckdeschel G: Decrease in nosocomial pneumonia in ventilated patients by selective oropharyngeal decontamination (SOD). *Intensive Care Med*. 1997;23:187-95.
S173. Aerdts SJ, van Dalen R, Clasener HA, Festen J, van Lier HJ, Vollaard EJ: Antibiotic prophylaxis of respiratory tract infection in mechanically ventilated patients. A prospective, blinded, randomized trial of the effect of a novel regimen. *Chest*. 1991;100:783-791
S174. Bergmans DC, Bonten MJ, Gaillard CA, et al Prevention of ventilator-associated pneumonia by oral decontamination: a prospective, randomized, double-blind, placebo-controlled study. *Am J Respir Crit Care Med* 2001;164:382-388
S175. Blair P, Rowlands BJ, Lowry K, Webb H, Armstrong P, Smilie J Selective decontamination of the digestive tract: a stratified, randomized, prospective study in a mixed intensive care unit. *Surgery* 1991;110:303-309
S176. Boland JP, Sadler DL, Stewart W, Wood DJ, Zerick W, Snodgrass KR. Reduction of nosocomial respiratory tract infections in the multiple trauma patients requiring mechanical ventilation by selective parenteral and enteral antisepsis regimen (SPEAR) in the intensive care. InXVII International Congress of Chemotherapy: 1991.
S177. Bonten MJ, Gaillard CA, Johanson Jr WG, Van Tiel FH, Smeets HG, Van Der Geest S, Stobberingh EE. Colonization in patients receiving and not receiving topical antimicrobial prophylaxis. *Am J Respir Crit Care Med.* 1994;150(5):1332-40.
S178. Cockerill FR, 3rd, Muller SR, Anhalt JP, et al. Prevention of infection in critically ill patients by selective decontamination of the digestive tract. *Ann Intern Med* 1992;117:545-53.
S179. de Jonge E, Schultz MJ, Spanjaard L, et al. Effects of selective decontamination of digestive tract on mortality and acquisition of resistant bacteria in intensive care: a randomised controlled trial. *Lancet* 2003; 362(9389): 1011-6.
S180. de Smet AMGA, Kluytmans JAJW, Cooper BS, Mascini EM, Benus RFJ, van der Werf TS, van der Hoeven JG, Pickkers P, Bogaers-Hofman D, van der Meer NJ, Bernards AT, Kuijper EJ, Joore JC, Leverstein-van Hall MA, Bindels AJ, Jansz AR, Wesselink RM, de Jongh BM, Dennesen PJ, van Asselt GJ, te Velde LF, Frenay IH, Kaasjager K, Bosch FH, van Iterson M, Thijsen SF, Kluge GH, Pauw W, de Vries JW, Kaan JA, Arends JP, Aarts LP, Sturm PD, Harinck HI, Voss A, Uijtendaal EV, Blok HE, Thieme Groen ES, Pouw ME, Kalkman CJ, Bonten MJ: Decontamination of the digestive tract and oropharynx in ICU patients. *N Engl J Med* 2009, 360:20–31.

S181. De Latorre FJ, Pont T, Ferrer A, Rosselló J, Palomar M, Planas M. Pattern of tracheal colonization during mechanical ventilation. *Am J Respir Crit Care Med*. 1995;152(3):1028-33.
S182. Georges B, Mazerolles M, Decun JF, Rouge P, Pomies S, Cougot P, Andrieu P, Virenque CH. Décontamination digestive sélective résultats d'une étude chez le polytraumatisé. *Reanimation Urgences*. 1994; 3(6):621-7.
S183. Hammond JM, Potgieter PD, Saunders GL, Forder AA: Double-blind study of selective decontamination of the digestive tract in intensive care. *Lancet* 1992, 340:5-9.
S184. Hammond JM, Potgieter PD: Long-term effects of selective decontamination on antimicrobial resistance. *Crit Care Med* 1995, 23:637-45.
S185. Jacobs S, Foweraker JE, Roberts SE. Effectiveness of selective decontamination of the digestive tract (SDD) in an ICU with a policy encouraging a low gastric pH. *Clin Intens Care*. 1992;3:52-8.
S186. Karvouniaris M, Makris D, Zygoulis P, Triantaris A, Xitsas S, Mantzarlis K, et al. Nebulised colistin for ventilator-associated pneumonia prevention. *Eur Respir J*. 2015;46:1732–9.
S187. Kerver AJ, Rommes JH, Mevissen-Verhage EA, Hulstaert PF, Vos A, Verhoef J, Wittebol P: Prevention of colonization and infection in critically ill patients: a prospective randomized study. *Crit Care Med* 1988, 16:1087-1093.
S188. Krueger WA, Lenhart FP, Neeser G, Ruckdeschel G, Schreckhase H, Eissner HJ, Forst H, Eckart J, Peter K, Unertl KE: Influence of combined intravenous and topical antibiotic prophylaxis on the incidence of infections, organ dysfunctions, and mortality in critically ill surgical patients: a prospective, stratified, randomized, double-blind, placebo-controlled clinical trial. *Am J Respir Crit Care Med* 2002, 166:1029-1037.
S189. Laggner AN, Tryba M, Georgopoulos A, Lenz K, Grimm G, Graninger W, Schneeweiss B, Druml W: Oropharyngeal decontamination with gentamicin for long-term ventilated patients on stress ulcer prophylaxis with sucralfate? *Wien Klin Wochenschr* 1994, 106:15-19.
S190. Ledingham I, Eastaway A, Mckay I, Alcock S, Mcdonald J, Ramsay G: Triple regimen of selective decontamination of the digestive tract, systemic cefotaxime, and microbiological surveillance for prevention of acquired infection in intensive care. *Lancet* 1988, 1:785-90.
S191. Lingnau W, Berger J, Javorsky F, et al: Selective intestinal decontamination in multiple trauma patients: Prospective, controlled trial. *J Trauma* 1997; 42: 687–94.
S192. McClelland P, Murray AE, Williams PS, van Saene HK, Gilbertson AA, Mostafa SM, Bone JM: Reducing sepsis in severe combined acute renal and respiratory failure by selective decontamination of the digestive tract. *Crit Care Med* 1990, 18:935-9.
S193. Palomar M, Alvarez-Lerma F, Jorda R, Bermejo B, Catalan Study Group of Nosocomial Pneumonia Prevention: Prevention of nosocomial infection in mechanically ventilated patients: selective digestive decontamination versus sucralfate. *Clin Intens Care.* 1997;8:228-235
S194. Pneumatikos I, Koulouras V, Nathanail C, Goe D, Nakos G: Selective decontamination of subglottic area in mechanically ventilated patients with multiple trauma. *Intensive Care Med* 2002;28:432-437
S195. Pugin J, Auckenthaler R, Lew DP, et al. Oropharyngeal decontamination decreases incidence of ventilator-associated pneumonia. A randomized, placebo-controlled, double-blind clinical trial. *JAMA* 1991;265:2704-10.
S196. Rocha LA, Martin MJ, Pita S, Paz J, Seco C, Margusino L, Villanueva R, Duran MT: Prevention of nosocomial infection in critically ill patients by selective decontamination of the digestive tract. A randomized, double blind, placebo-controlled study. *Intensive Care Med*. 1992;18:398-404
S197. Sanchez Garcia M, Cambronero Galache JA, Lopez Diaz J, Cerda Cerda E, Rubio Blasco J, Gomez Aguinaga MA, Nunez Reiz A, Rogero Marin S, Onoro Canaveral JJ, Sacristan del Castillo JA: Effectiveness and cost of selective decontamination of the digestive tract in critically ill intubated patients. A randomized, double-blind, placebo-controlled, multicenter trial. *Am J Respir Crit Care Med* 1998, 158:908-916.
S198. Stoutenbeek CP, van Saene HKF, Zandstra DF. Prevention of multiple organ failure by selective decontamination of the digestive tract in multiple trauma patients. In: Faist E, Baue AE, Schildberg FW, editors. The immune consequences of trauma, shock and sepsisdmechanisms and therapeutic approach. Lengerich: Pabst Science Publishers; 1996. p. 1055-1066.
S199. Stoutenbeek CP, van Saene HKF, Little RA, Whitehead A: The effect of selective decontamination of the digestive tract on mortality in multiple trauma patients: a multicenter randomized controlled trial. *Intensive Care Med*. 2007;33:261-270
S200. Verwaest C, Verhaegen J, Ferdinande P, Schetz M, Van den Berghe G, Verbist L, Lauwers P: Randomized, controlled trial of selective digestive decontamination in 600 mechanically ventilated patients in a multidisciplinary intensive care unit. *Crit Care Med.* 1997;25:63-71
S201. Wiener J, Itokazu G, Nathan C, Kabins SA, Weinstein RA: A randomized, double-blind, placebo-controlled trial of selective digestive decontamination in a medical-surgical intensive care unit. *Clin Infect Dis*. 1995;20:861-867

S202. Winter R, Humphreys H, Pick A, MacGowan AP, Willatts SM, Speller DC. A controlled trial of selective decontamination of the digestive tract in intensive care and its effect on nosocomial infection. *J Antimicrob Chemother*. 1992;30(1):73-87.
S203. Wittekamp BH, Plantinga NL, Cooper BS, Lopez-Contreras J, Coll P, Mancebo J, Wise MP, Morgan MP, Depuydt P, Boelens J, Dugernier T. Decontamination strategies and bloodstream infections with antibiotic-resistant microorganisms in ventilated patients: a randomized clinical trial. *JAMA*. 2018.
S204. Wood GC, Boucher BA, Croce MA, Hanes SD, Herring VL, Fabian TC. Aerosolized ceftazidime for prevention of ventilator-associated pneumonia and drug effects on the proinflammatory response in critically ill trauma patients. *Pharmacotherapy*. 2002;22:972-982.
S205. Garbino J, Lew DP, Romand JA, Hugonnet S, Auckenthaler R, Pittet D: Prevention of severe Candida infections in non-neutropenic, high-risk, critically ill patients: a randomized, double-blind, placebo-controlled trial in patients treated by selective digestive decontamination. *Intensive Care Med* 2002;28:1708-1717
S206. Garbino J, Pichard C, Pichna P, Pittet D, Lew D, Romand J (2004) Impact of enteral versus parenteral nutrition on the incidence of fungal infections: a retrospective study in ICU patients on mechanical ventilation with selective digestive decontamination. *Clinical Nutrition* 23;705-710.
S207. Oudhuis GJ, Bergmans DC, Dormans T, Zwaveling JH, Kessels A, Prins MH, Verbon A: Probiotics versus antibiotic decontamination of the digestive tract: infection and mortality. *Intensive Care Med* 2011, 37:110-117.
S208. van der Geest PJ, Mohseni M, Linssen J, Duran S, de Jonge R, Groeneveld AB. The intensive care infection score–a novel marker for the prediction of infection and its severity. *Crit care*. 2016;20(1):1-8.
S209. Van Meenen DM, Van Der Hoeven SM, Binnekade JM, De Borgie CA, Merkus MP, Bosch FH, Endeman H, Haringman JJ, Van Der Meer NJ, Moeniralam HS, Slabbekoorn M. Effect of on-demand vs routine nebulization of acetylcysteine with salbutamol on ventilator-free days in intensive care unit patients receiving invasive ventilation: a randomized clinical trial. *JAMA*. 2018;319(10):993-1001.
S210. Veelo DP, Bulut T, Dongelmans DA, Korevaar JC, Spronk PE, Schultz MJ. The incidence and microbial spectrum of ventilator-associated pneumonia after tracheotomy in a selective decontamination of the digestive tract-setting. *J Infection.* 2008;56(1):20-6.
S211. Safdar N, Dezfulian C, Collard HR, Saint S. Clinical and economic consequences of ventilator-associated pneumonia: a systematic review. *Crit Care Med* 2005;33(10):2184-93.
S212. Melsen WG, Rovers MM, Bonten MJM: Ventilator-associated pneumonia and mortality: A systematic review of observational studies. *Crit Care Med* 2009, 37:2709–2718.
S213. Agrafiotis M, Siempos II, Ntaidou TK, Falagas ME. Attributable mortality of ventilator-associated pneumonia: a meta-analysis. *Internat J Tuberculosis Lung Dis*. 2011;15(9):1154-63.
S214. Pileggi C, Mascaro V, Bianco A, Nobile CG, Pavia M. Ventilator bundle and its effects on mortality among ICU patients: a meta-analysis. *Crit Care Med*. 2018;46(7):1167-74.
S215. Toews_I, George_AT, Peter_JV, Kirubakaran_R, Fontes_LES, Ezekiel_JPB, Meerpohl_JJ. Interventions for preventing upper gastrointestinal bleeding in people admitted to intensive care units. Cochrane Database of Systematic Reviews 2018, Issue 6. Art. No.: CD008687.
S216. Lewis SR, Schofield-Robinson OJ, Alderson P, Smith AF. Enteral versus parenteral nutrition and enteral versus a combination of enteral and parenteral nutrition for adults in the intensive care unit. Cochrane Database of Systematic Reviews 2018, Issue 6. Art. No.: CD012276.
S217. Padilla PF, Martínez G, Vernooij RW, Urrútia G, i Figuls MR, Cosp XB. Early enteral nutrition (within 48 hours) versus delayed enteral nutrition (after 48 hours) with or without supplemental parenteral nutrition in critically ill adults. Cochrane Database of Systematic Reviews. 2019(10).
S218. Alkhawaja S, Martin C, Butler RJ, Gwadry-Sridhar F. Post-pyloric versus gastric tube feeding for preventing pneumonia and improving nutritional outcomes in critically ill adults. Cochrane Database of Systematic Reviews. 2015(8).
S219. Solà I, Benito S. Closed tracheal suction systems versus open tracheal suction systems for mechanically ventilated adult patients. Cochrane Database of Systematic Reviews 2007, Issue 4. Art. No.: CD004581.
S220. Gillies D, Todd DA, Foster JP, Batuwitage BT. Heat and moisture exchangers versus heated humidifiers for mechanically ventilated adults and children. Cochrane Database of Systematic Reviews 2017, Issue 9. Art. No.: CD004711.
S221. Wang L, Li X, Yang Z, Tang X, Yuan Q, Deng L, Sun X. Semi-recumbent position versus supine position for the prevention of ventilator-associated pneumonia in adults requiring mechanical ventilation. Cochrane Database of Systematic Reviews 2016, Issue 1. Art. No.: CD009946.
S222. Tokmaji G, Vermeulen H, Müller MCA, Kwakman PHS, Schultz MJ, Zaat SAJ. Silver-coated endotracheal tubes for prevention of ventilator-associated pneumonia in critically ill patients. Cochrane Database of Systematic Reviews 2015, Issue 8. Art. No.: CD009201.
S223. Bo L, Li J, Tao T, Bai Y, Ye X, Hotchkiss RS, Kollef MH, Crooks NH, Deng X. Probiotics for preventing ventilator-associated pneumonia. Cochrane Database of Systematic Reviews 2014, Issue 10. Art. No.: CD009066.

S224. Hua F, Xie H, Worthington HV, Furness S, Zhang Q, Li C. Oral hygiene care for critically ill patients to prevent ventilator-associated pneumonia. Cochrane Database of Systematic Reviews 2016, Issue 10. Art. No.: CD008367.
S225. Zhao T, Wu X, Zhang Q, Li C, Worthington HV, Hua F. Oral hygiene care for critically ill patients to prevent ventilator-associated pneumonia. Cochrane Database of Systematic Reviews 2020, Issue 12. Art. No.: CD008367.
S226. Liberati A, D'Amico R, Pifferi S, Torri V, Brazzi L, Parmelli E. Antibiotic prophylaxis to reduce respiratory tract infections and mortality in adults receiving intensive care. Cochrane Database of Systematic Reviews 2009, Issue 4. Art. No.: CD000022.
S227. Minozzi S, Pieri S, Brazzi L, Pecoraro V, Montrucchio G, D'Amico R. Topical antibiotic prophylaxis to reduce respiratory tract infections and mortality in adults receiving mechanical ventilation. Cochrane Database of Systematic Reviews 2021, Issue 1. Art. No.: CD000022.

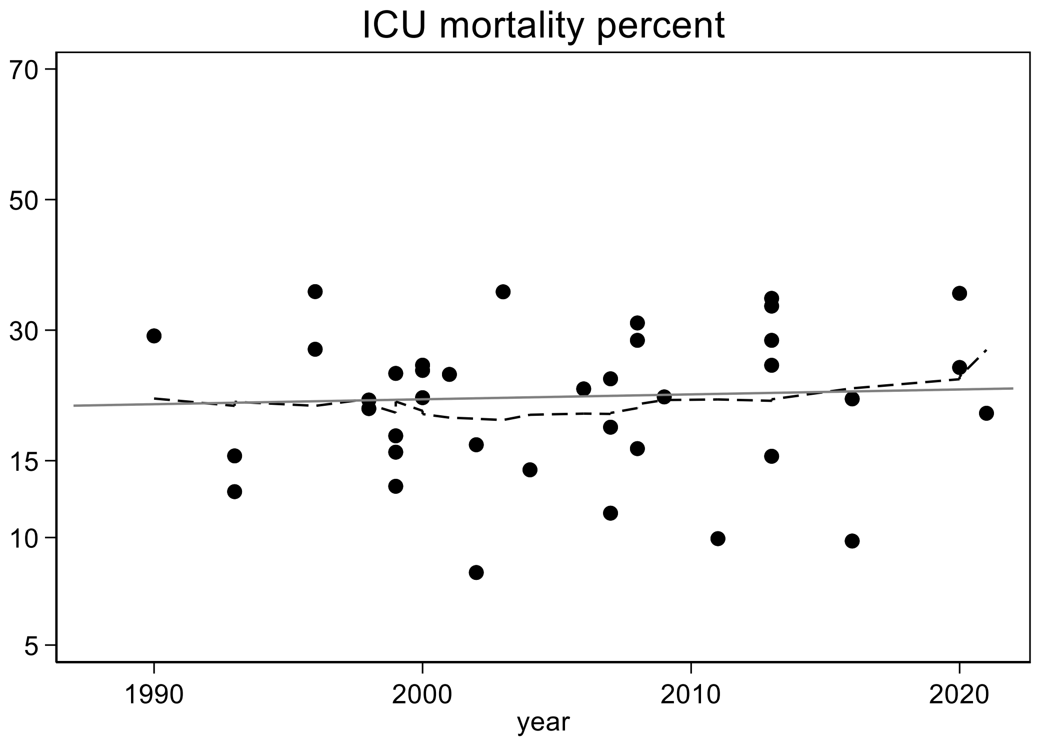

Fig S1 A LOESS (locally weighted scatter plot smoothing) plot (Broken line) derived using a 0.5 bandwidth together with a linear regression line (solid line) of mortality incidence (logit scale) versus year of study publication among observational groups

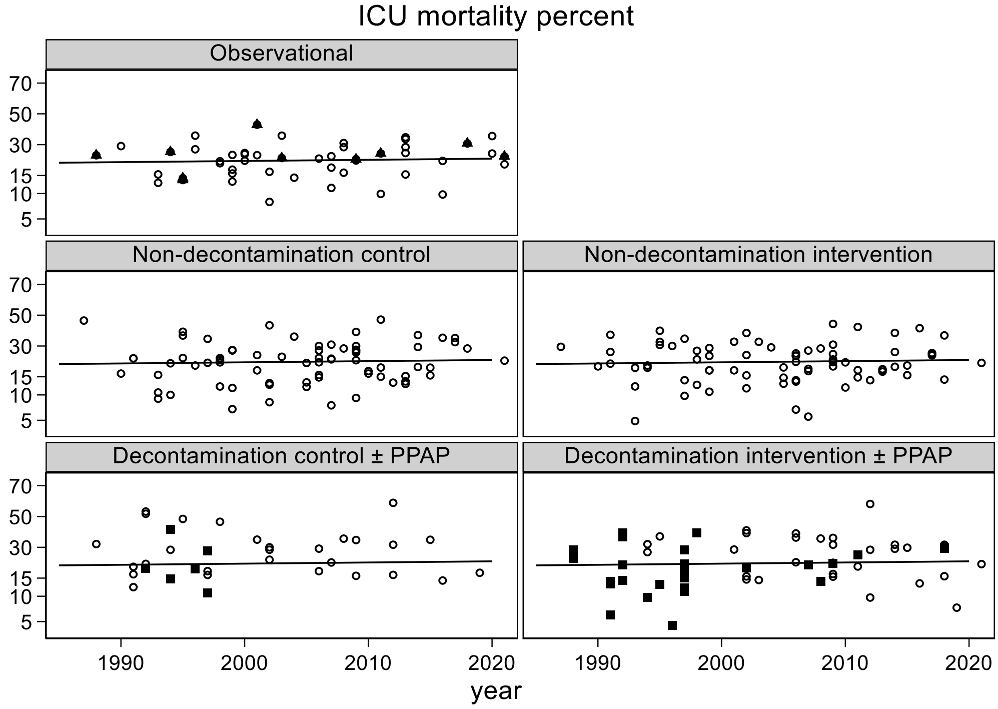

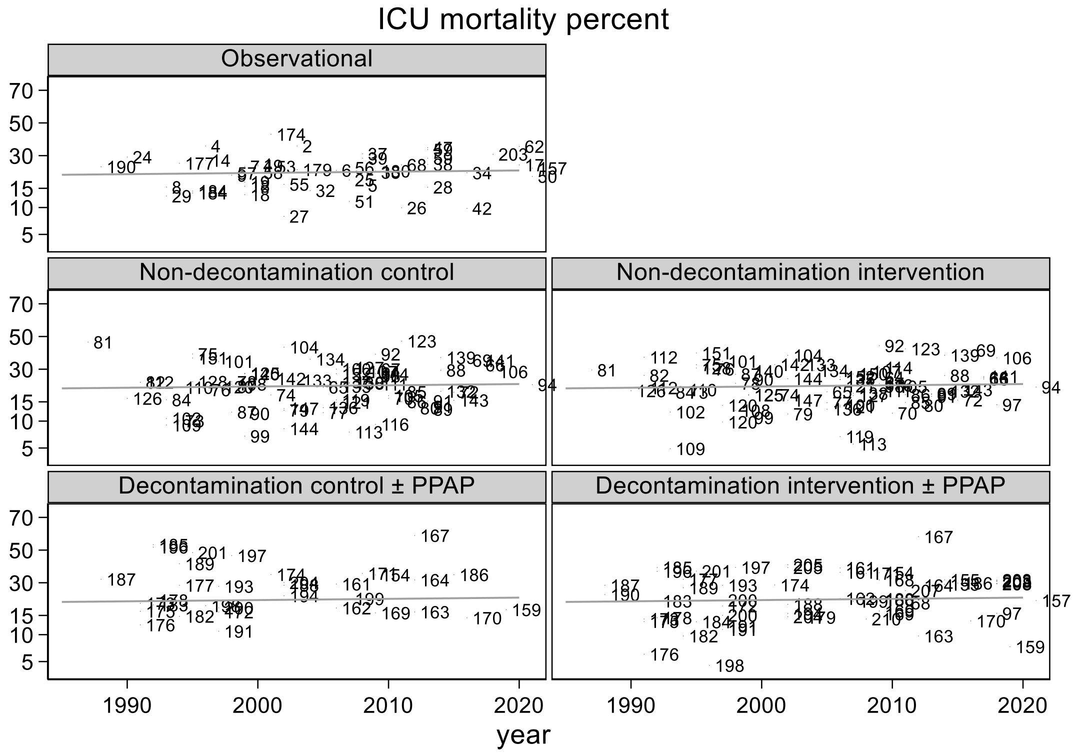

Fig S2 Scatter plot and linear regression of ICU mortality [mortality censured at day 21 or less] incidence versus year of study publication for groups from studies of infection prevention interventions. The linear regression line in each plot, derived using the observational groups, increased non-significantly versus year of study publication (slope is +1.2 percentage points per decade; 95% confidence interval -1.9 to +4.3; p = 0.64) and serves as a benchmark for all plots. The equivalent plot for late mortality is shown as Fig S3. Note the y-axis is a logit scale. The lower plot shows the study reference number of each group. [Symbols; ▲= non-concurrent control groups; ■= groups receiving PPAP; ○ = all other groups; non-concurrent control groups are included as observational]

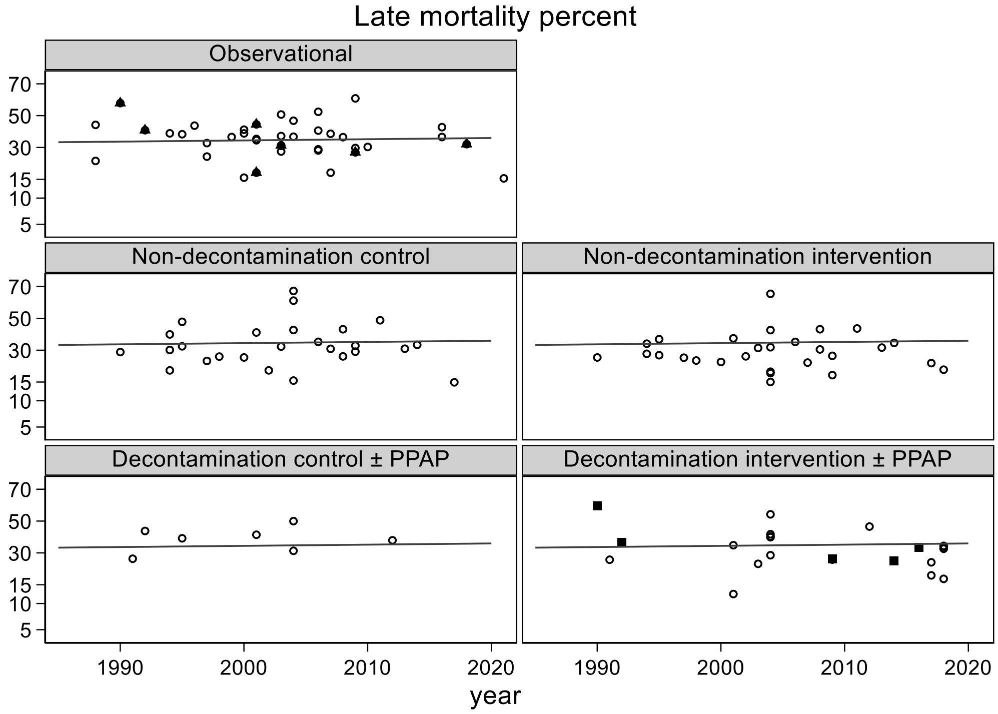

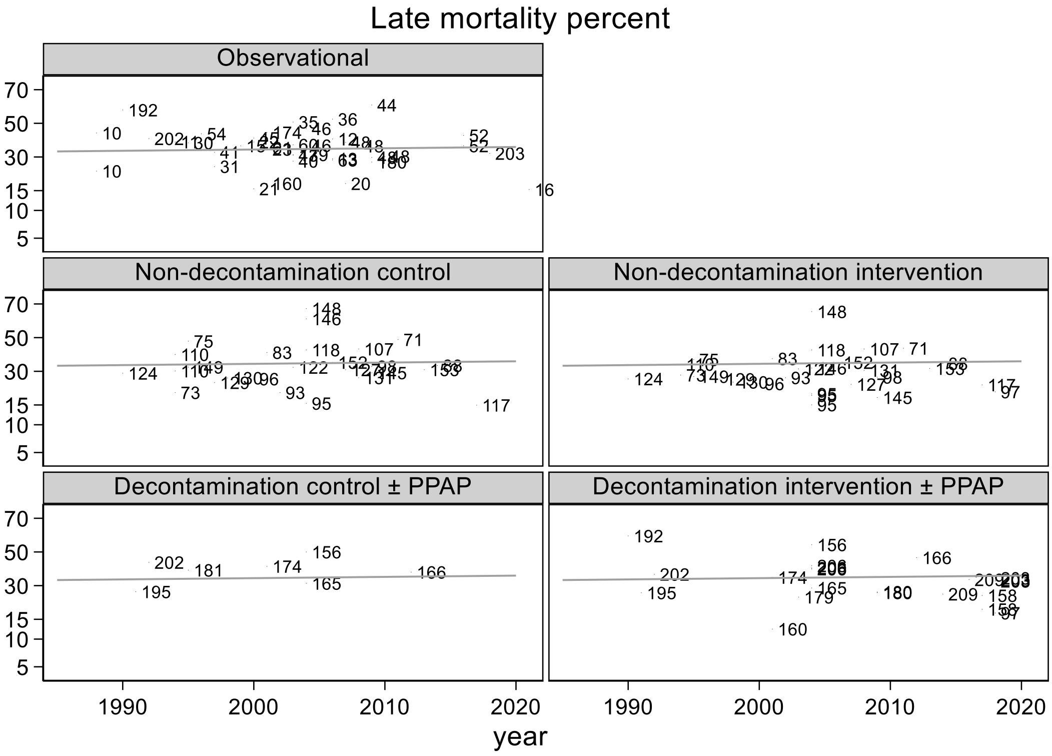

Fig S3 Scatter plot and linear regression of ICU mortality [mortality censured at day 21 or less] incidence versus year of study publication for groups from studies of infection prevention interventions. The linear regression line in each plot, derived using the observational groups, increased non-significantly versus year of study publication (slope is +1.2 percentage points per decade; 95% confidence interval -1.9 to +4.3; p = 0.64) and serves as a benchmark for all plots. The equivalent plot for ICU mortality is shown as Fig S2. Note the y-axis is a logit scale. The lower plot shows the study reference number of each group. [Symbols; ▲= non-concurrent control groups; ■= groups receiving PPAP; ○ = all other groups; non-concurrent control groups are included as observational]

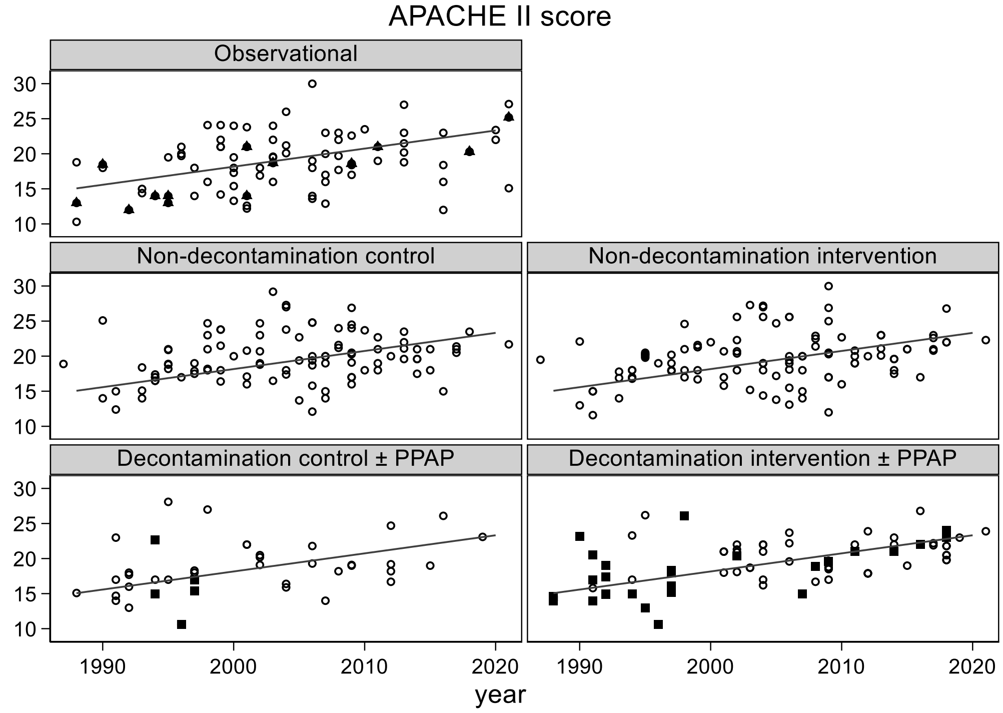

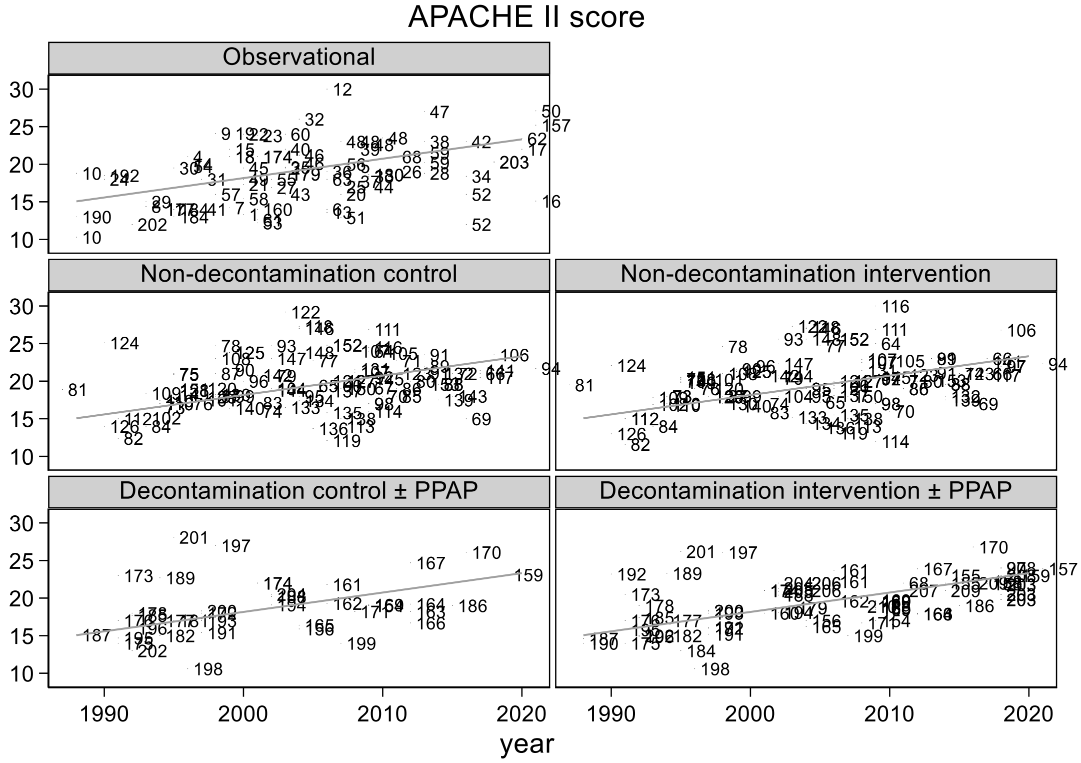

Fig S4 Scatter plot and linear regression of group mean APACHE II score versus year of study publication for groups from studies of infection prevention interventions. The linear regression line in each plot, derived using the observational groups, increased versus year of study publication (slope is +2.2 points per decade point; 95% confidence interval +1.1 to +3.3; p = 0.001) and serves as a benchmark for all plots. Note the y-axis is a logit scale. The lower plot shows the study reference number of each group. [Symbols; ▲= non-concurrent control groups; ■= groups receiving PPAP; ○ = all other groups; non-concurrent control groups are included as observational]

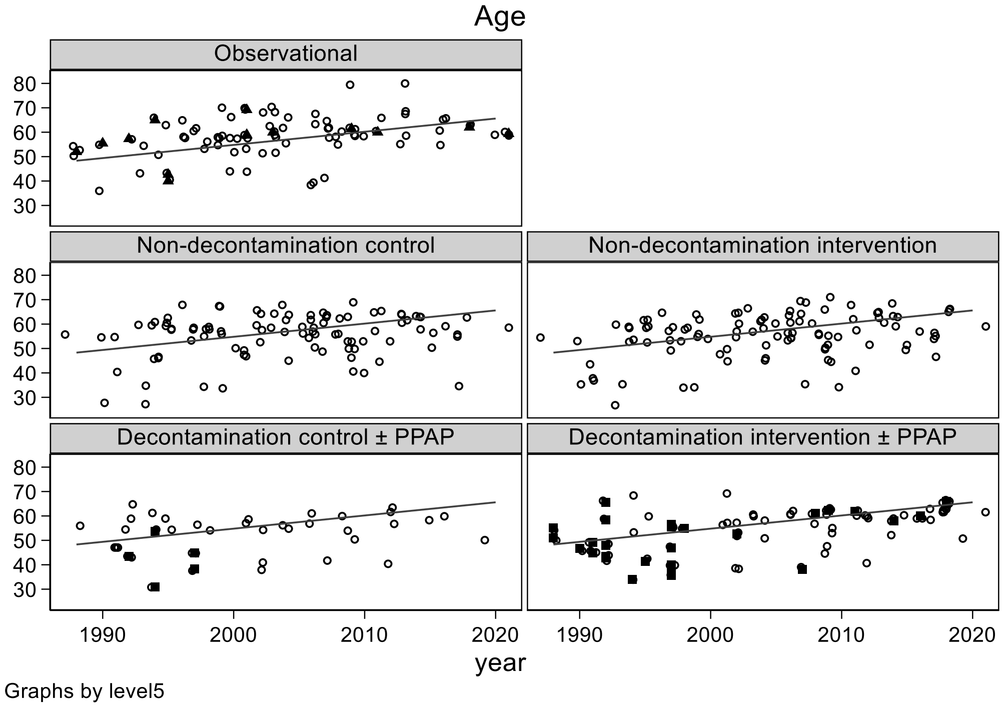

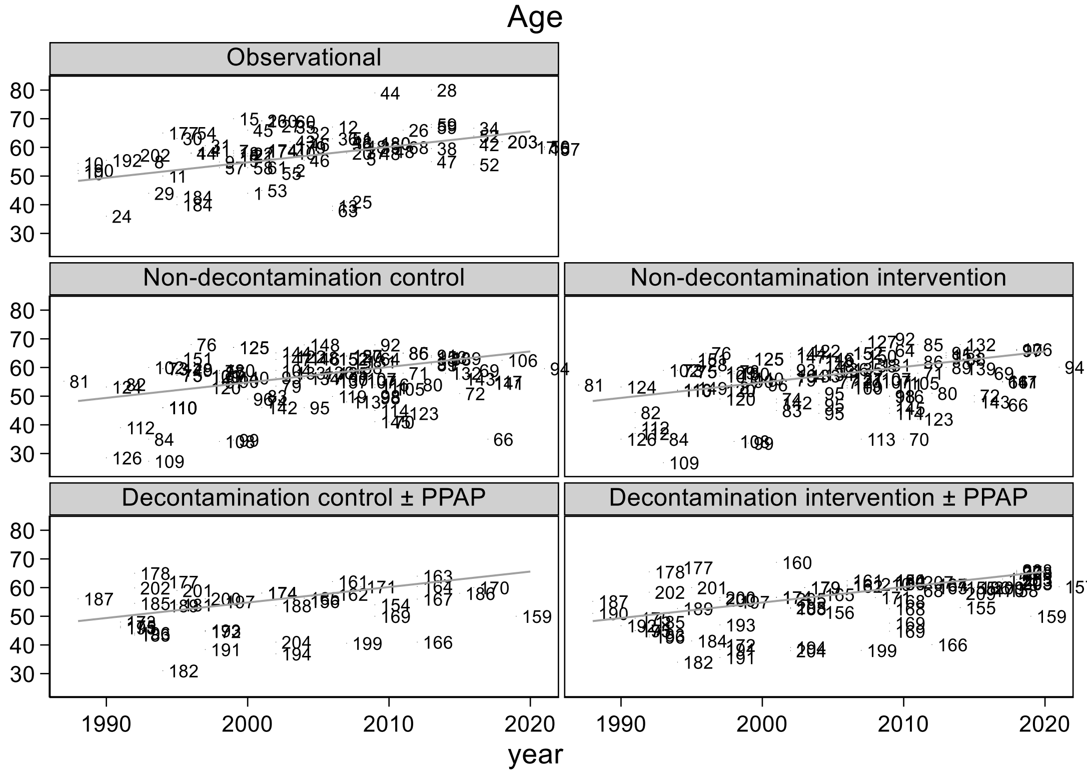

Fig S5 Scatter plot and linear regression of group mean mean age versus year of study publication for groups from studies of infection prevention interventions. The linear regression line in each plot, derived using the observational groups, increased versus year of study publication (slope is +3.9 points per decade point; 95% confidence interval +1.7 to +6.1; p = 0.001) and serves as a benchmark for all plots. Note the y-axis is a logit scale. The lower plot shows the study reference number of each group. [Symbols; ▲= non-concurrent control groups; ■= groups receiving PPAP; ○ = all other groups; non-concurrent control groups are included as observational]

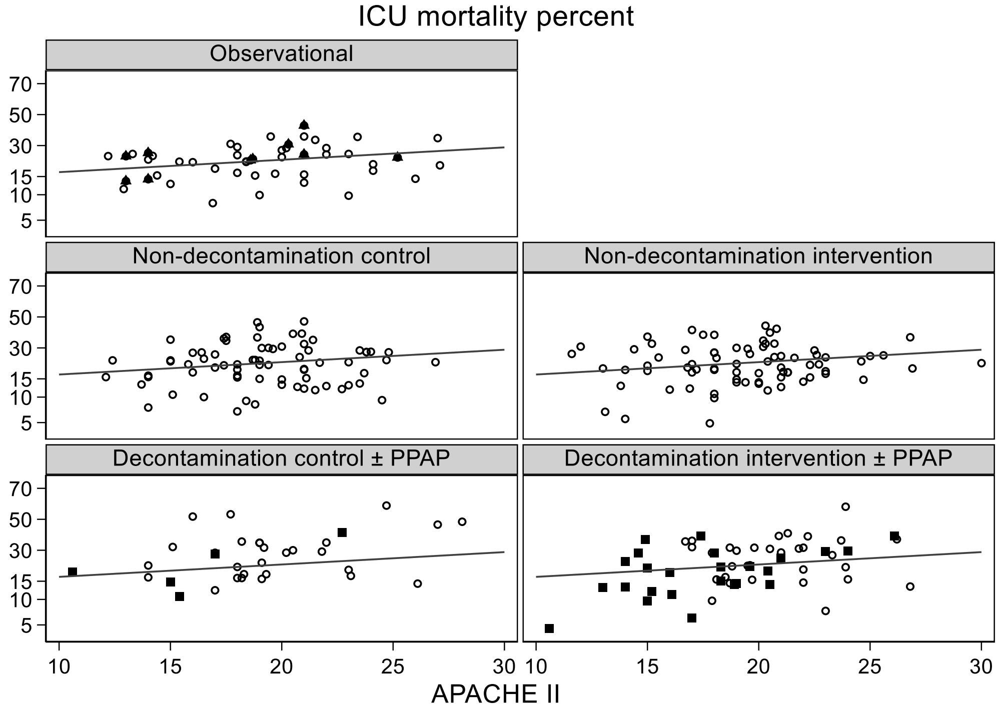

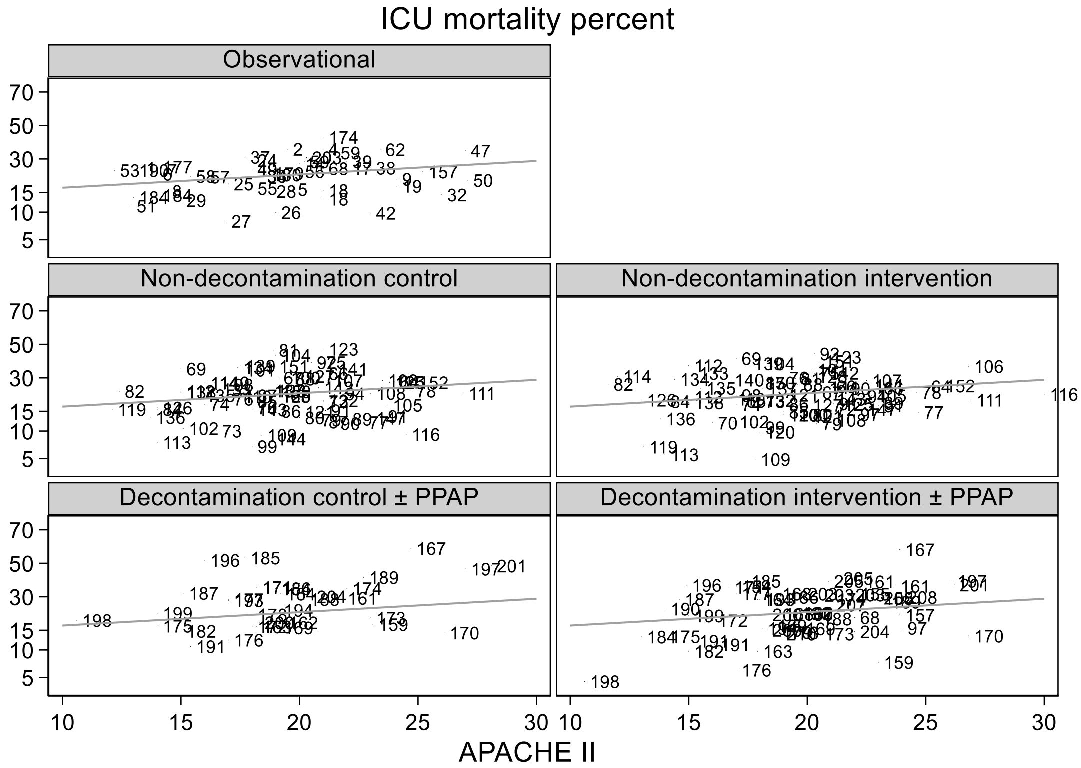

Fig S6 Scatter plot and linear regression of ICU mortality [mortality censured at day 21 or less] incidence versus group mean APACHE II score for groups from studies of infection prevention interventions. The linear regression line in each plot, derived using the observational groups, increased versus year of study publication (slope is +0.7 percentage points per APACHE II score point; 95% confidence interval +0.2 to +1.2; p = 0.003) and serves as a benchmark for all plots. Note the y-axis is a logit scale. The lower plot shows the study reference number of each group. [Symbols; ▲= non-concurrent control groups; ■= groups receiving PPAP; ○ = all other groups; non-concurrent control groups are included as observational]

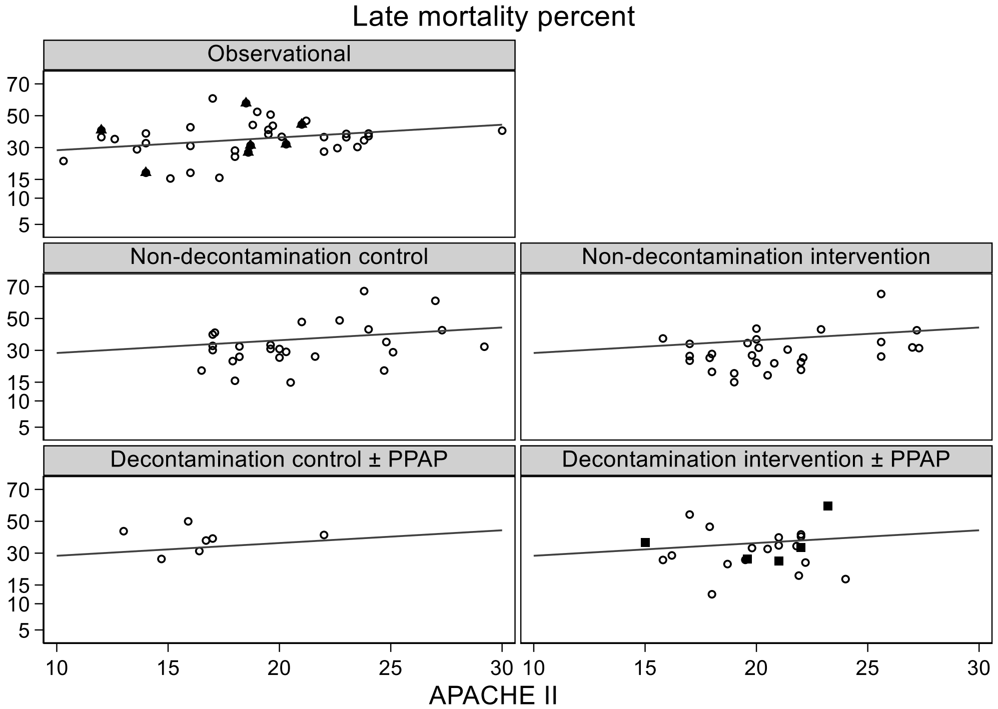

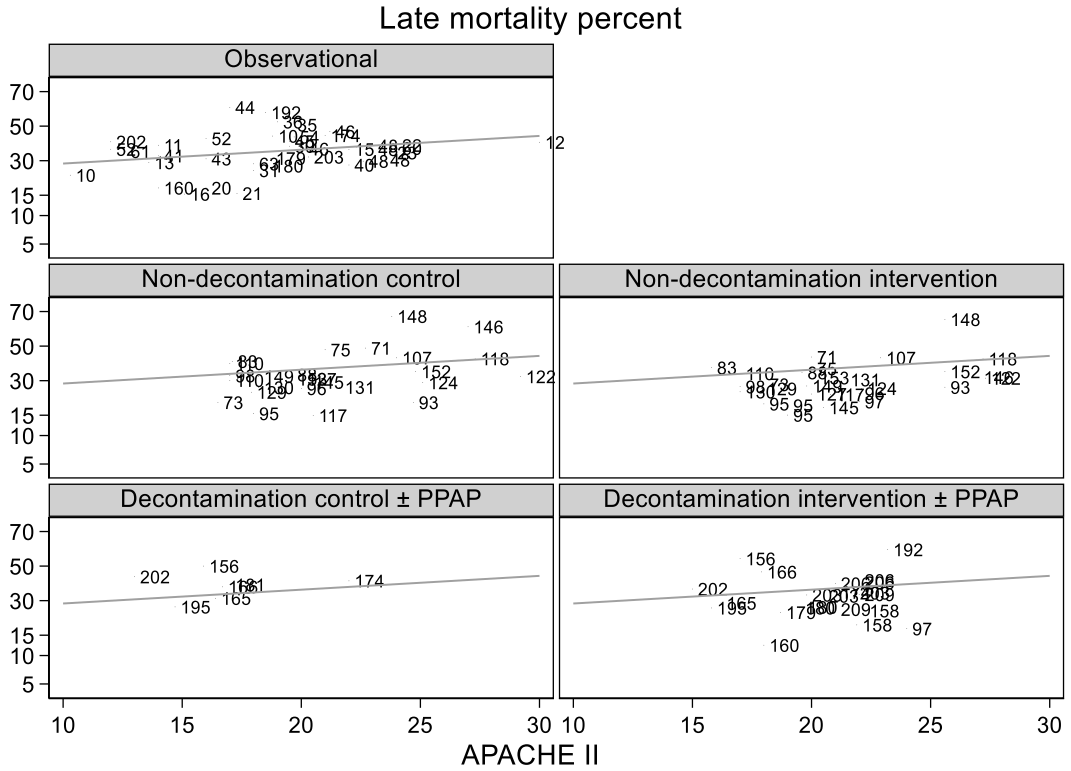

Fig S7 Scatter plot and linear regression of late mortality [mortality censured after day 21] incidence versus group mean APACHE II score for groups from studies of infection prevention interventions. The linear regression line in each plot, derived using the observational groups, increased versus year of study publication (slope is +0.7 percentage points per APACHE II score point; 95% confidence interval +0.2 to +1.2; p = 0.003) and serves as a benchmark for all plots. Note the y-axis is a logit scale. The lower plot shows the study reference number of each group. [Symbols; ▲= non-concurrent control groups; ■= groups receiving PPAP; ○ = all other groups; non-concurrent control groups are included as observational]
